# Efficient Solution-Phase Dipeptide Synthesis Using Titanium Tetrachloride and Microwave Heating

**DOI:** 10.3390/ijms25179729

**Published:** 2024-09-08

**Authors:** Palmira Alessia Cavallaro, Marzia De Santo, Rocco Marinaro, Emilia Lucia Belsito, Angelo Liguori, Antonella Leggio

**Affiliations:** Department of Pharmacy, Health and Nutritional Sciences, University of Calabria, Via P. Bucci, 87036 Arcavacata di Rende, Italy; alessia.cavallaro@unical.it (P.A.C.); marzia.desanto@unical.it (M.D.S.); rocco.marinaro@unical.it (R.M.); emilialucia.belsito@unical.it (E.L.B.); angelo.liguori@unical.it (A.L.)

**Keywords:** titanium tetrachloride, Lewis acid, dipeptides, microwaves, peptide synthesis, condensing agent

## Abstract

Microwaves have been successfully employed in the Lewis acid titanium tetrachloride-assisted synthesis of peptide systems. Dipeptide systems with their amino function differently protected with urethane protecting groups have been synthesized in short periods of time and with high yields. The formation of the peptide bond between the two reacting amino acids was achieved in pyridine by using titanium tetrachloride as a condensing agent and heating the reaction mixture with a microwave reactor. The reaction conditions are compatible with amino acids featuring various side chains and different protecting groups on both the amino function and side chains. Additionally, the substrates retain their chiral integrity after reaction.

## 1. Introduction

Amide linkage is one of the most widespread chemical bonds in organic molecules, drugs, and biologically active compounds, including peptides and proteins [1,2]. The amide or peptide bond is of fundamental importance for the stability and structural characteristics of all known proteins [3].

Peptides play a crucial role in biology and have gained significant importance in the biomedical field. Their growing importance in medicine is due to their versatility, specificity, and therapeutic potential, offering advantages like high specificity, lower toxicity, and the ability to target complex molecular structures [4]. Recent advancements have led to the discovery of new peptides with various therapeutic benefits [5]. Peptide-based drugs such as leuprolide for prostate cancer and liraglutide for type 2 diabetes are now available on the market [6]. Peptides are also being used in vaccine development [7] and targeted therapies to deliver drugs to specific sites, block cell surface receptors, and interfere with intracellular protein interactions [8]. Recently approved peptides include Setmelanotide, which helps manage weight in genetic obesity cases by activating the melanocortin 4 receptor [9], Trofinetide, designed to treat Rett syndrome [10], and Voclosporin, a peptide-based calcineurin inhibitor, used as an immunosuppressant for lupus nephritis [11]. Peptides are also proving to be valuable in cancer treatment and diagnostic imaging, like Lutetium Lu-177 Vipivotide Tetraxetan, a radiolabeled peptide targeting the prostate-specific membrane antigen (PSMA) for treating metastatic prostate cancer [12] and Gallium Ga-68 Gozetotide, a peptide-based imaging agent used in PET scans to visualize PSMA in prostate cancer patients [13]. 

The synthesis of organic molecules incorporating amide bonds, including peptides, has been extensively explored. Numerous studies have reported a variety of synthetic approaches in this regard [14,15,16,17,18,19]. 

Recent technological advancements have introduced a fully automated, flow-based method for solid-phase polypeptide synthesis (AFPS). This method combines the flexibility of solid-phase peptide synthesis (SPPS) with significantly faster processing times. AFPS improves peptide production by drastically reducing the time needed for amide bond formation in standard Fmoc peptide synthesis and offers better control compared to traditional manual or automated methods [20]. In line with these advancements, Hurevich et al. developed a high-yield, cost-efficient, affordable, and fast peptide synthetic process using high-temperature fast-stirring peptide synthesis (HTFSPS). Their approach allowed them to accelerate solid-phase synthesis, proving the effective use of overhead stirring and heating without an excess of reagent, making it possible to produce a large number of peptides in a short period of time [21]. 

The activation of amino acid carboxylic function is a key step in peptide synthesis. It is generally performed through the conversion of *N*-terminal amino acids into reactive acylating intermediates (acyl chlorides, anhydrides, and activated esters) using activating reagents [22,23,24,25,26]. However, activation and coupling steps often face significant challenges, including the use of toxic and expensive reagents [27,28], the generation of hard-to-isolate byproducts [29], and the risk of racemization at the activated carboxyl residues [30]. These issues underscore the ongoing need for more efficient coupling reagents and improved methods.

Recently, metal-based reagents for amide bond formation have attracted significant attention due to their efficiency and reactivity [31,32,33,34]. Titanium (IV)-based reagents have gained prominence in organic synthesis. Their ability to coordinate with the carbonyl oxygen atom and their versatility in transforming various functional groups make them valuable tools in peptide synthesis [33,34,35,36,37,38,39,40]. We investigated the use of titanium tetrachloride (TiCl_4_) for peptide bond formation and developed a new solution-phase method for synthesizing dipeptides [41,42]. In this method, TiCl_4_ serves as the condensing agent in a pyridine-buffered medium at 40 °C. This approach produced various *N*-protected dipeptides within approximately 5 h. However, the 5-hour reaction time can be a limitation for synthesizing longer peptide chains. To address this, we explored the use of microwave irradiation to accelerate the process. Microwave (MW) heating, in fact, allows us to enhance reaction yields and decrease reaction times by directly interacting with reagents and solvent, offering a faster and more efficient method of heating the reaction mixtures compared to conventional methods [43,44,45,46,47,48]. Microwave irradiation not only accelerates the reaction but also offers a more sustainable and efficient approach compared to traditional heating. However, despite these benefits, the use of microwave irradiation in solution-phase peptide synthesis remains underexplored.

Previous studies have demonstrated the potential of microwave-assisted methods. For instance, Jain et al. developed a rapid microwave-assisted protocol for dipeptide synthesis using DIC/HONB in DMF, achieving a 65% yield with no racemization [49]. Building on this, the same research group reported the first solution-phase peptide synthesis conducted in neat water using TBTU/HOBt/DIEA under microwave irradiation in 2013 [50]. 

In this study, we present a novel method for solution-phase peptide synthesis that combines TiCl_4_ as a condensing agent with microwave heating, enabling the efficient formation of dipeptide systems with urethane protecting groups on the *N*-terminal amino function.

## 2. Results and Discussion

Titanium tetrachloride (TiCl_4_) was employed as a condensing agent for the solution-phase synthesis of aspartame, a commercially used artificial sweetener, as well as a series of dipeptides protected on the amino function with *tert*-butoxycarbonyl (Boc), fluorenylmethoxycarbonyl (Fmoc) and benzyloxycarbonyl (Z) groups [42]. The reactions, performed in pyridine as solvent, were completed within 5–6 h.

Although the reaction conditions were mild, with a reaction temperature of 40 °C employed to avoid side reactions, reaction times were only acceptable (5–6 h). Furthermore, the procedure required the use of excess TiCl_4_ (2 eq) relative to the amino acid substrates (1 eq). 

The evaluation of these aspects has led to the belief that the use of a microwave reactor could obviate these drawbacks, improving the efficiency of the process and consequently further enhancing the synthetic utility of the reaction. Therefore, we decided to carry out peptide bond formation under microwave irradiation and experiment with an equimolar ratio (1:1) between the amino acid substrate and TiCl_4_. 

**Scheme 1:**
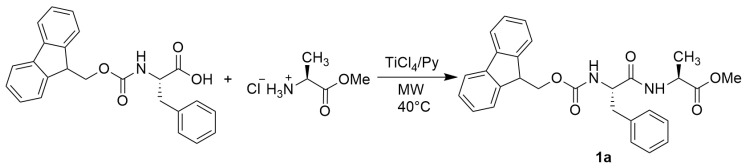
Synthesis of *N*-Fmoc-Phe-Ala-OMe (**1a**).

Preliminarily, the reaction was tested by synthesizing the dipeptide *N*-Fmoc-L-Phe-L-Ala-OMe (**1a**), chosen as model system, to evaluate the progress of the reaction and identify the best reaction conditions. Alanine methyl ester hydrochloride (1 mmol) dissolved in pyridine (3 mL) was treated with *N*-Fmoc-L-phenylalanine (1 mmol) and TiCl_4_ (1mmol); then, the reaction mixture was stirred and subjected to microwave irradiation at 40 °C, with the reaction monitored through TLC analysis (Scheme 1). Pyridine was chosen as the solvent based on previous studies [42], which demonstrated its effectiveness in enhancing the performance of the TiCl_4_ reagent system.

To identify the best microwave power required for the reaction, we tested a range of power settings from 50 W to 250 W for synthesizing compound **1a** (Table 1). The results of these experiments revealed that running the reaction at 250 W resulted in high yields of product **1a** and significantly shorter reaction times compared to the lower power settings of 50 W and 100 W. 

The reaction conducted under microwave irradiation at 250 W produced the dipeptide **1a** in just 20 min with a high 90% yield, using an equimolar ratio of TiCl_4_ to the starting *N*-Fmoc-protected amino acid.

Furthermore, prolonged reaction times did not improve product yield. However, these conditions offered a notable time improvement compared to the previous conventional heating method, which demanded a reaction time of 5–6 h. The dipeptide **1a** was recovered by co-evaporating pyridine with toluene, then filtering the crude reaction product suspended in chloroform through a silica gel column, using chloroform as the eluent.

In light of these results, we performed a direct comparison with the reaction conducted under conventional heating, using the same temperature (40 °C) and the same molar ratio between TiCl_4_ and the amino acid substrate (1:1).

Specifically, the reaction between *N*-Fmoc-phenylalanine (1 mmol) and alanine methyl ester hydrochloride (1 mmol) in the presence of TiCl_4_ (1 mmol) was carried out in pyridine using an oil bath maintained at 40 °C. After 1 h, the reaction mixture yielded only 33% of dipeptide **1a**. After 5 h, a significant amount of the starting substrates (about 50%) remained in the reaction mixture. 

**Scheme 2:**
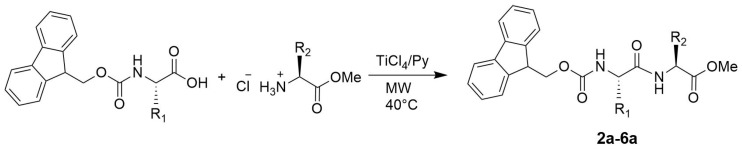
Synthesis of *N*-Fmoc-dipeptide methyl esters **2a**–**6a**.

Encouraged by the successful microwave-assisted synthesis of dipeptide **1a**, we applied the same protocol to other *N*-Fmoc-protected amino acids to assess the reproducibility and effectiveness of the reaction (Scheme 2, Table 2).

The TiCl_4_-assisted reaction between *N*-Fmoc-L-Tyr(OtBu)-OH and glycine methyl ester hydrochloride efficiently produced ester **3a** in a high yield, as shown in Table 2. Similarly, the coupling of *N*-Fmoc-L-Cys(Bzl)OH with L-alanine methyl ester hydrochloride yielded the dipeptide *N*-Fmoc-L-Cys(Bzl)-L-Ala-OMe (**6a**) in 35 min with a 70% yield (Table 2). These results demonstrate that the reaction conditions effectively preserve the acid-labile protecting groups on the amino acid side chains.

All the obtained *N*-Fmoc-dipeptides (**1a**–**6a**) were characterized by ^1^H NMR and ^13^C NMR analyses. The condensation of *N*-Fmoc-protected amino acids with amino acid methyl ester hydrochlorides was successfully achieved, with all reactions demonstrating favorable yields and significantly reduced reaction times. 

The use of pyridine as a solvent effectively addresses the issue of releasing the amino function of amino acid methyl ester from its hydrochloride, as it is present in a large excess. Lastly, it is noteworthy that a 1:1 molar ratio of titanium tetrachloride to amino acid was consistently employed in each reaction.

These experiments demonstrate that microwave-driven reactions in TiCl_4_-assisted dipeptide synthesis are significantly faster than those conducted under conventional heating at the same temperature. This suggests that microwaves might have unique effects that enhance reaction rates beyond what standard heating can achieve. 

A potential issue arises from the difficulty in accurately determining the temperature inside the microwave cavity using an IR sensor. IR sensors only monitor the temperature on the external surface of the reaction vessel, which may not accurately reflect the actual internal reaction temperature. To assess potential non-thermal effects, precise temperature control is crucial. The optimal choice is an internal temperature sensor, such as an optical fiber probe, which can be directly placed into the reaction solution providing accurate measurements [47]. Moreover, maintaining a homogeneous and properly stirred reaction mixture is essential for reliable results.

When the microwave reactor was set to 40 °C, temperature fluctuations were observed. The IR sensor recorded temperatures ranging from 40 to 50 °C, while the fiber-optic probe detected a range of 40 to 60 °C. These variations are likely due to the constant microwave power applied until the set temperature is reached, followed by a feedback loop that modulates the power to maintain the temperature. Notably, the fiber-optic sensor revealed that the actual internal reaction temperature was higher than the temperature recorded by the IR sensor. 

To compare the microwave-assisted reaction at 40 °C with conventional heating at 60 °C, we adjusted the conventional heating temperature to match the maximum temperature measured by the fiber-optic probe during microwave irradiation. This adjustment accounts for the approximate 10 °C difference observed between the IR and fiber-optic sensors. The two experiments were conducted simultaneously to facilitate a direct comparison between the microwave-driven (40 °C) and conventional heating reactions (60 °C) for synthesizing the dipeptide Fmoc-Leu-Ile-OMe (**5a**). Both reactions were performed under identical conditions, including temperature, reagent concentration, and solution volume, in identical sealed reaction vessels. The molar ratios of TiCl_4_ (1 mmol) to the amino acid substrates, Fmoc-isoleucine (1 mmol) and leucine methyl ester hydrochloride (1 mmol), were consistent with those used for the model dipeptide Fmoc-Phe-Ala-OMe (**1a**). 

Initially, the solvent (pyridine) is heated to 60 °C using conventional heating with an oil bath, with the temperature inside the reaction vessel continuously monitored by a Vertex VTF digital thermoregulator. In the microwave-initiated reaction, the solvent temperature was initially set to 40 °C, but it fluctuated between 40 °C and 60 °C, as measured by an optic fiber probe placed within the reaction mixture. Once the solvent in both reactions reached the target temperature, the reagents were simultaneously added, and the mixtures were vigorously stirred using a magnetic stirrer to ensure temperature homogeneity throughout the reaction [47,51]. 

In the microwave-heated reaction, thermal conditions within the reaction mixture are uniform, with measurements taken at different points consistently showing temperature fluctuations between 40 and 60 degrees Celsius. In contrast, the temperature in the conventionally heated reaction remains stable at 60 degrees Celsius. Both reactions were monitored using thin-layer chromatography (TLC) with a chloroform–methanol solvent system (90:10 *v*/*v*). After approximately 40 min, the microwave-driven reaction was complete, while the conventional heating reaction was still ongoing. At this point, both reactions were stopped and processed identically. The microwave-assisted reaction yielded the expected product, Fmoc-Leu-Ile-OMe (**5a**, Table 2), with a 70% yield, while the conventional heating method produced only 20% of the product.

The process began by heating the solvent to the target temperature. Once reached, the reagents were added simultaneously to both systems, maintaining a nearly constant temperature throughout. This indicates that differences in heating rates cannot account for the variations in the reaction progress. Additionally, no overheating was observed in the microwave-assisted reaction, as the process was conducted well below the solvent’s boiling point. Therefore, the increased rate of the microwave-assisted reaction cannot be attributed to temperature effects, as described by the Arrhenius equation (k = Ae − Ea/BT), which relates reaction rates to temperature. Instead, microwaves may accelerate the reaction by affecting the frequency factor (A) in the Arrhenius equation, by inducing rapid rotational motion in polar reactants and intermediates and increasing the frequency of effective molecular collisions. This enhanced molecular agitation facilitates the condensation reaction, leading to a faster overall reaction rate.

To further demonstrate the protocol’s versatility, it was applied to the synthesis of dipeptide systems, employing acid-labile groups like Boc and Z to protect the α-amino function. 

**Scheme 3:**
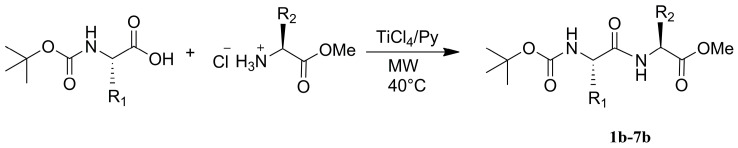
Synthesis of *N*-Boc-protected dipeptide methyl esters **1b**–**7b**.

The synthesis of *N*-Boc-dipeptides was conducted in a microwave reactor, employing the same reaction conditions used for the *N*-Fmoc-protected substrates (Scheme 3).

Even employing Boc-protected amino acids, the reaction proceeds rapidly, reaching completion within 20–40 min. After the reaction, the Boc-dipeptide systems **1b**–**7b** were efficiently recovered by the fast filtration of the reaction mixture suspended in chloroform through a silica column, with yields ranging from 70% to 94% (Table 3).

The analysis of the Boc-protected dipeptides was performed using nuclear magnetic resonance spectroscopy (^1^H NMR and ^13^C NMR) and gas chromatography/mass spectrometry (GC/MS (EI)).

*N*-Boc-arginine, with the guanidine group’s nitrogen atoms protected with the acid-labile benzyloxycarbonyl (Z) group, was reacted with alanine methyl ester hydrochloride under the previously established conditions. Following approximately 40 min, the dipeptide *N*-Boc-Arg(Z)_2_-OH (**6b**) was obtained with an 83% yield (Table 3) and high purity, as confirmed by ^1^H and ^13^C NMR analyses. The spectroscopic data confirmed the stability of both the Z group on the side chain and the Boc group on the α-amino function, as no deprotection occurred under the reaction conditions.

**Figure 1:**
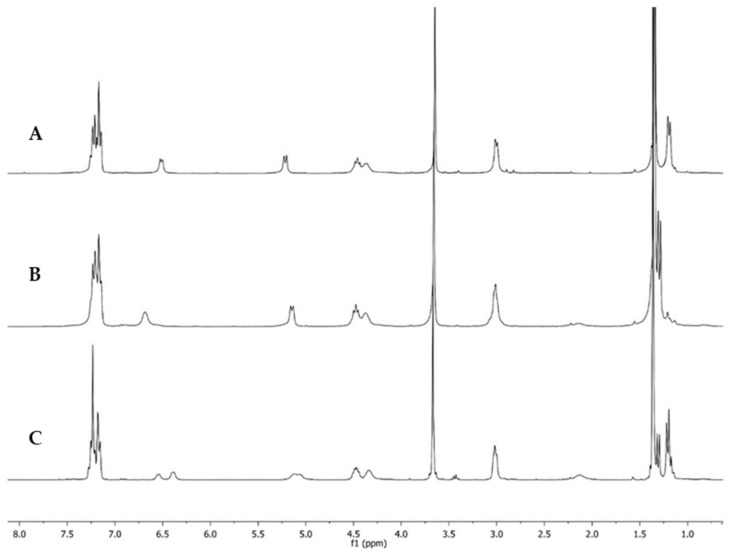
^1^H NMR spectra of *N*-Boc dipeptides: (**A**) *N*-Boc-L-Phe-D-Ala-OCH_3_ (**2b**); (**B**) *N*-Boc-D-Phe-D-Ala-OCH_3_ (**3b**); (**C**) mixture consisting of 60% **2b** and 40% **3b**.

The reaction conditions also preserved the optical purity at the chiral centers of the precursors, as evidenced by ^1^H NMR analysis of a couple of diastereoisomeric dipeptides (**2b** and **3b**). The examination of the ^1^H NMR spectra for both single epimers of *N*-Boc-L-Phe-D-Ala-OMe (Figure 1A) and *N*-Boc-D-Phe-D-Ala-OMe (Figure 1B) showed signals consistent with a single diastereomer, indicating minimal epimerization within the detection limits of the NMR technique (Figure 1). Distinct chemical shifts were observed for the signals originating from the amide NH protons (6.51 and 6.69 ppm) and alanine side chain CH_3_ protons (1.19 and 1.30 ppm) in the two diastereoisomers. These differences were clearly resolved in the ^1^H NMR spectrum of a mixture containing **2b** and **3b**, specifically prepared for this analytical purpose (Figure 1C).

**Scheme 4:**
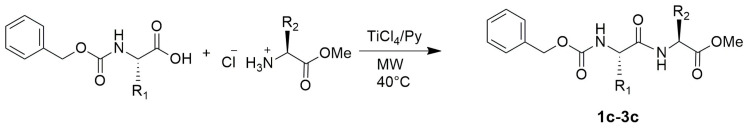
Solution-phase synthesis of *N*-Z-protected dipeptide methyl esters **1c**–**3c**.

Moreover, the application of microwave irradiation in the TiCl_4_-assisted coupling reaction also produced notable results in the synthesis of dipeptides protected on the terminal amino function with the benzyloxycarbonyl (Z) protecting group (Scheme 4). The reactions to form the *N*-Z-protected dipeptides (Table 4) were completed within just 30–35 min, with product yields ranging from 65% to 81%. The structures of the Z-protected dipeptides were confirmed using proton (^1^H) and carbon-13 (^13^C) nuclear magnetic resonance (NMR) spectroscopy, as well as gas chromatography/mass spectrometry (GC/MS). These analyses also confirmed that the Z protecting group remained intact on the terminal amino function under the conditions of the microwave-assisted coupling procedure.

## 3. Materials and Methods

### 3.1. General Information

Reagents were commercially available with analytical grade and used as purchased without further purification. Solvents were purified following standard laboratory techniques and freshly distilled before use. 

Reactions were conducted in a CEM Discover single-mode microwave reactor equipped with an infrared temperature sensor (IR). Additionally, a fiber-optic probe provided by the manufacturer was used to directly monitor the internal reaction temperature within a 10 mL sealed reaction vessel. 

The microwave reactions were carried out at 40 °C in 10 mL glass vials (obtained from CEM) sealed with silicone/PTFE caps. The pressure release limit was set to 250 psi, and the power was set to 250 W, with the microwave instrument dynamically controlling the applied power. 

The reaction mixtures were stirred magnetically and monitored by thin-layer chromatography (TLC) using silica gel 60-F254 pre-coated glass plates. The TLC spots were visualized under a UV lamp (254 nm) and by spraying with 0.2% ninhydrin in ethanol, followed by charring after elution.

^1^H and ^13^C NMR spectra were recorded on a Bruker Avance 300 instrument at 300 MHz and 75 MHz, respectively. Spectroscopic analysis was performed at 293 K on diluted solutions of each compound by using CDCl_3_ as solvent. Chemical shifts (δ) are reported in ppm, while coupling constants (J) are reported in Hertz (Hz). 

GC-MS analyses were performed using an Agilent GC-6890/MSD-5973 system with electronic ionization detectors and an HP-5MS capillary column (30 m × 0.25 mm, 5% diphenyl 95% dimethylpolysiloxane). The mass detector operated in an electron impact ionization mode (EI/MS) with an electron energy of 70 eV. The injection port temperature was set to 250 °C. The oven temperature program started at 70 °C with a 2 min hold, then ramped to 280 °C at a rate of 20 °C/min, and held for 10 min. ^1^H, ^13^C NMR, and GC/MS spectra are available in the Supporting Materials.

### 3.2. General Procedure for the Synthesis of N-Protected Dipeptides

α-Amino acid methyl ester hydrochloride (1 mmol) is first dissolved in 3 mL of anhydrous pyridine in a 10 mL reaction vessel equipped with a magnetic stir bar. Immediately after, *N*-protected-α-amino acid (1 mmol) and TiCl_4_ (1 mmol) are added to the solution.

The reaction mixture is stirred magnetically for 1–2 min while monitoring the pH, adjusting with additional pyridine to maintain near-neutral conditions if necessary. The vessel is then sealed with a PTFE cap and irradiated in the microwave reactor.

The reaction progress is monitored by TLC analysis using a chloroform–methanol (90:10, *v*/*v*) solvent system and is completed in 20–40 min. Afterward, pyridine is removed by co-evaporation with toluene. The resulting crude product is suspended in chloroform and purified by column chromatography on silica gel, which also contains layers of NaHCO₃ and NaHSO_4_ separated by silica gel, using chloroform as the mobile phase. The evaporation of chloroform under reduced pressure yields the corresponding *N*-protected dipeptides with yields ranging from 65% to 94%.

***N-Fmoc-L-Phe-L-Ala-OMe (1a)***

**^1^H NMR** (300 MHz, CDCl_3_) δ 7.78 (d, *J* = 7.5 Hz, 2H, ArH), 7.55 (t, *J* = 6.4 Hz, 2H, ArH), 7.42 (t, *J* = 7.4 Hz, 2H, ArH), 7.37–7.09 (*m*, 5H, ArH), 6.43 (s_broad_, 1H, CONH), 5.45 (s_broad_, 1H, OCONH), 4.66–4.41 (*m*, 3H, CH_2_Fmoc, CHCOOMe), 4.33 (*m*, 1H, CHCONH), 4.20 (t, *J* = 6.9 Hz, 1H, CHFmoc), 3.72 (s, 3H, OCH_3_), 3.20–3.96 (m, 2H, CH_2_Ph), 1.35 (d, *J* = 7.1 Hz, 3H, CH_3_). **^13^C NMR** (75 MHz, CDCl_3_) δ 172.77, 170.31, 155.89, 143.75, 141.30, 136.22, 129.39, 128.70, 127.75, 127.10, 125.03, 120.01, 67.11, 56.03, 52.48, 48.18, 47.12, 38.61, 18.31. 

Analytical data matched those previously reported in the literature [52].

***N-Fmoc-Gly-L-Ala-OMe (2a)***

**^1^H NMR** (300 MHz, CDCl_3_) δ 7.73 (d, J = 7.5 Hz, 2H, ArH), 7.56 (d, J = 7.3 Hz, 2H, ArH), 7.47–7.19 (*m*, 4H, ArH), 6.80 (d_broad_, J = 6.4 Hz, 1H, CONH), 5.72 (t_broad_, J = 5.4 Hz, 1H, OCONH), 4.78–4.48 (*m*, 1H, CHCOOMe), 4.37 (d, J = 7.0 Hz, 2H, CH_2_Fmoc), 4.19 (t, J = 7.0 Hz, 1H, CHFmoc), 4.05–3.81 (*m*, 2H, CH_2_CONH), 3.69 (s, 3H, OCH_3_), 1.37 (d, J = 7.1 Hz, 3H, CH_3_CH). **^13^C NMR** (75 MHz, CDCl_3_) δ: 173.85, 169.23, 157.23, 144.31, 141.86, 128.26, 127.64, 125.72, 120.71, 67.90, 53.06, 48.73, 47.68, 44.88, 18.82.

***N-Fmoc-L-Tyr(tBu)-Gly-OMe (3a)***

**^1^H NMR** (300 MHz, CDCl_3_) 7.76 (d, J = 7.4 Hz, 2H, ArH), 7.59–7.49 (*m*, 2H, ArH), 7.40 (t, J = 7.2 Hz, 2H, ArH), 7.32 (d, J = 7.2 Hz, 2H, ArH), 7.17–7.03 (*m*, 2H, ArH), 6.91 (d, J = 7.4 Hz, 2H, ArH), 6.41 (s_broad_, 1H, OCONH), 5.50 (s_broad_, 1H, CONH), 4.55–4.28 (*m*, 3H, CH_2_Fmoc, CHCONH), 4.18 (t, *J* = 6.8 Hz, 1H, CHFmoc), 4.07–3.89 (*m*, 2H, CH_2_COOMe), 3.71 (s, 3H, OCH_3_), 3.11–2.99 (*m* 2H, CH_2_-Tyr), 1.31 (s, 9H, C(CH_3_)_3_). **^13^C NMR** (75 MHz, CDCl_3_) δ: 170.75, 169.57, 154.59, 143.74, 141.31, 129.71, 127.73, 127.09, 125.02, 124.29, 119.97, 78.39, 67.09, 56.04, 52.31, 47.16, 41.17, 37.69, 28.82.

***N-Fmoc-L-Leu-Gly-OMe (4a)***

**^1^H NMR** (300 MHz, CDCl_3_) δ 7.76 (d, *J* = 7.5 Hz, 2H, ArH), 7.65–7.53 (*m*, 2H, ArH), 7.38 (d, *J* = 7.4 Hz, 2H, ArH), 7.35–7.27 (*m*, 2H, ArH), 6.89 (s_broad_, 1H, CONH), 5.55 (s_broad_, 1H, OCONH), 4.50–4.37 (*m*, 2H, CH_2_Fmoc), 4.34–4.26 (*m*, 1H, CHCONH), 4.20 (t, J = 6.9 Hz, 1H, CHFmoc) 4.02 (*m*, 2H, CH_2_COOMe, 3.71 (s, 3H, OCH_3_), 1.80–1.46 (*m*, 3H, CH_2_CH), 1.04–0.88 (*m*, 6H, CH(CH_3_)_2_). **^13^C NMR** (75 MHz, CDCl_3_) δ 172.76, 170.19, 156.35, 143.79, 141.30, 127.74, 127.09, 125.07, 120.00, 67.01, 53.32, 52.38, 47.15, 41.25, 29.73, 24.65, 22.95, 21.93.

Analytical data matched those previously reported in the literature [53].

***N-Fmoc-L-Leu-L-Ile-OMe (5a)***

**^1^H-NMR** (300 MHz, CDCl_3_) δ 7.77 (d, J = 7.5 Hz, 2H, ArH), 7.59 (d, J = 7.5 Hz, 2H, ArH), 7.49–7.20 (*m*, 4H, ArH), 6.58 (d, J = 8.4 Hz, 1H, CONH), 5.36 (d, J = 8.4 Hz, 1H, OCONH), 4.58 (dd, J = 8.4, 5.1 Hz, 1H, CHCOOMe), 4.50–4.35 (*m*, 2H, CH_2_-Fmoc), 4.33–4.16 (*m*, 2H, CH-Fmoc, CHCONH), 3.73 (s, 3H, OCH_3_), 1.98–1.75 (*m*, 2H, CHCH_3_, CH_2_CH(CH_3_)_2_), 1.74–1.59 (*m*, 2H, CH_2_CH(CH_3_)_2_), 1.47–1.33 (*m*, 1H, CH_2_CH_3_), 1.23–1.08 (*m*, 1H, CH_2_CH_3_), 1.07–0.74 (*m*, 12H, CHCH_3_, CH_2_CH_3_, CH_2_CH(CH_3_)_2_). **^13^C NMR** (75 MHz, CDCl_3_) δ: 172.05, 171.93, 155.62, 143.80, 141.31, 127.71, 127.06, 125.02, 119.97, 67.27, 56.46, 53.62, 52.02, 47.19, 41.29, 37.86, 25.14, 24.67, 22.86, 15.40, 11.48.

***N-Fmoc-L-Cys(Bzl)-L-Ala-OMe (6a)***

**^1^H NMR** (300 MHz, CDCl_3_) δ 7.68 (d, 2H, J = 7.5 Hz, ArH), 7.58–7.46 (*m*, 2H, ArH), 7.42–7.09 (*m*, 9H, ArH), 6.72 (s_broad_, 1H, CONH), 5.57 (s_broad_, 1H, OCONH), 4.47 (*m*, 1H, CHCOOMe), 4.41–4.26 (*m*, 2H, CH_2_-Fmoc), 4.25–4.15 (*m*, 1H, CHCONH), 4.14–4.10 (*m*, 1H, CH-Fmoc), 3.75–3.56 (*m*, 5H, SCH_2_Ph, OCH_3_), 2.80 (*m*, 1H, CHCH_2_S), 2.67 (*m*, 1H, CHCH_2_S), 1.32 (d, 3H, J = 7.2 Hz, CH_3_).

***N-Boc-L-Phe-L-Ala-OMe (1b)***

**^1^H NMR** (300 MHz, CDCl_3_) δ 7.42–7.11 (*m*, 5H, ArH), 6.63 (s_broad_, 1H, CONH), 5.14 (s_broad_, 1H, OCONH), 4.54 (*m*, 1H, CHCOOMe), 4.40 (*m*, 1H, CHCH_2_Ph), 3.72 (s, 3H, OCH_3_), 3.07 (d, J = 6.2 Hz, 2H, CH_2_Ph), 1.41 (s, 9H, C(CH_3_)_3_), 1.35 (d, J = 7.2 Hz, 3H, CHCH_3_).**^13^C NMR** (75 MHz, CDCl_3_) δ 173.49, 171.45, 155.98, 137.10, 129.95, 129.16, 127.50, 80.76, 56.13, 48.69, 48.59, 38.95, 28.82, 18.89. **GC-MS (EI)** *m*/*z* (% rel.): 294 (7), 263 (1), 234 (8), 131 (8), 120 (82), 91 (22), 57 (100).

Analytical data matched those previously reported in the literature [52].

***N-Boc-L-Phe-D-Ala-OMe (2b)***

**^1^H NMR** (300 MHz, CDCl_3_) δ 7.24–7.14 (*m*, 5H, ArH), 6.51 (d, J = 7.0 Hz, 1H, CONH), 5.22 (d, J = 8.0 Hz, 1H, OCONH), 4.45 (*m*, 1H, CHCOOMe), 4.35 (*m*, 1H, CHCH_2_Ph), 3.65 (s, 3H, OCH_3_), 3.01 (d, J = 6.5 Hz, 2H, CH_2_Ph), 1.34 (s, 9H, C(CH_3_)_3_), 1.19 (d, J = 6.9 Hz, 3H, CHCH_3_).**^13^C NMR** (75 MHz, CDCl_3_) δ 173.05, 170.80, 155.40, 136.68, 129.35, 128.55, 126.87, 80.07, 55.64, 52.53, 47.87, 38.77, 28.23, 17.96. **GC-MS (EI)** *m*/*z* (% rel.): 350 (3), 294 (9), 233 (15), 131 (14), 120 (100), 91 (27), 57 (67).

***N-Boc-D-Phe-D-Ala-OMe (3b)***

**^1^H NMR** (300 MHz, CDCl_3_) δ 7.17 (t, *J* = 9.4 Hz, 5H, ArH), 6.69 (s, 1H, CONH), 5.14 (d, *J* = 7.6 Hz, 1H, OCONH), 4.48 (t, *J* = 6.8 Hz, 1H, CHCOOMe), 4.36 (*m*, 1H, CHCH_2_Ph), 3.66 (s, 3H, OCH_3_), 3.01 (s, 2H, CH_2_Ph), 1.35 (s, 9H, C(CH_3_)_3_), 1.30 (d, *J* = 7.2 Hz, 3H, CHCH_3_). **^13^C NMR** (75 MHz, CDCl_3_) δ 172.89, 170.97, 155.41, 136.61, 129.40, 128.61, 126.88, 80.11, 55.51, 52.53, 48.10, 38.52, 28.23, 18.19. **GC-MS (EI)** *m*/*z* (% rel.): 233 (10), 131 (14), 120 (90), 91 (25), 57 (100).

***N-Boc-L-Ile-L-Leu-OMe (4b)***

**^1^H-NMR** (300 MHz, CDCl_3_) δ 6.35 (d, *J* = 7.6 Hz, 1H, CONH), 5.08 (d, *J* = 8.9 Hz, 1H, OCONH), 4.57 (*m*, 1H, CHCOOMe), 3.89 (*m*, 1H, CHCONH), 3.68 (s, 3H, OCH_3_), 1.81 (s_broad_, 1H, CHCH_3_), 1.66–1.45 (*m*, 4H, CH_2_CH_3_), CH_2_CH), 1,38 (s, 9H, C(CH_3_)_3_), 1.22–1.08 (*m*, 1H, CH(CH_3_)_2_), 0.99–0.89 (*m*, 12H, CH(CH_3_)_2_, CH_2_(CH_3_)_2_). **^13^C NMR** (75 MHz, CDCl_3_) δ 173.16, 171.54, 155.78, 79.86, 59.13, 52.38, 50.61, 41.37, 37.09, 28.27, 24.73, 22.80, 21.80, 15.41, 11.32. **GC-MS (EI)** *m*/*z* (% rel.): 302 (3), 285 (6), 186 (19), 172 (2), 144 (3), 130 (91), 86 (100), 57 (48).

Analytical data matched those previously reported in the literature [53].

***N-Boc-L-Leu-L-Val-OMe (5b)***

**^1^H NMR** (300 MHz, CDCl_3_) δ 6.57 (d_broad_, *J* = 8.0 Hz, 1H, CONH), 4.87 (d, *J* = 8.2 Hz, 1H, OCONH), 4.51 (dd, *J* = 8.0, 4.9 Hz, 1H, CHCOOMe), 4.09 (m, 1H, CHCONH), 3.70 (s, 3H, OCH_3_), 2.21–2.07 (d, *J* = 4.6 Hz, 2H, CH_2_CH(CH_3_)_2_, CH(CH_3_)_2_), 1.73–1.58 (*m*, 2H, CH_2_CH(CH_3_)_2_), 1.41 (s, 9H, C(CH_3_)_3_), 0.97–0.83 (*m* 12H, CH(CH_3_)_2_, CH_2_CH(CH_3_)_2_). **^13^C NMR** (75 MHz, CDCl_3_) δ 172.39, 172.19, 155.56, 80.13, 56.99, 56.91, 53.11, 40.61, 31.30, 28.29, 27.80, 24.67, 22.87, 22.09, 18.93, 17.59. **GC-MS (EI)** *m*/*z* (% rel.): 344 (1), 271 (5), 186 (15), 158 (1), 130 (100), 86 (90), 72 (14), 57 (53).

***N-Boc-L-Arg(Z)_2_-L-Ala-OMe (6b)***

**^1^H NMR** (300 MHz, CDCl_3_) δ 9.56–9.18 (*m*, 2H, NHZ, NH), 7.39–7.23 (*m*, 10H, ArH), 6.91 (d, *J* = 7.0 Hz, 1H, CONH), 5.57 (d, *J* = 8.4 Hz, 1H, OCONH), 5.22–5.05 (*m*, 4H, CH_2_Ph), 4.43 (*m*, 1H, CHCOOMe), 4.25 (*m*, 1H, CHCONH), 4.12–3.75 (*m*, 2H, CH_2_NH), 3.63 (s, 3H, OCH_3_), 1.82–1.55 (*m*, 4H, CHCH_2_CH_2_CH_2_NH), 1.39 (s, 9H, C(CH_3_)_3_), 1.16 (d, *J* = 7.2 Hz, 3H, CHCH_3_). **^13^C NMR** (75 MHz, CDCl_3_) δ 172.90, 171.76, 163.61, 160.70, 155.85, 136.67, 134.63, 128.82, 128.47, 127.92, 127.71, 79.84, 69.00, 67.00, 53.60, 52.35, 48.02, 44.04, 28.84, 28.33, 24.59, 17.67.

***N-Boc-L-Phe-L-Leu-OMe (7b)***

**^1^H NMR** (300 MHz, CDCl_3_) δ 7.44–7.09 (*m*, 5H, ArH), 6.36 (d, J = 7.9 Hz, 1H, CONH), 5.08 (d, J = 8.0 Hz, 1H, OCONH), 4.57 (*m*, 1H, CHCOOMe), 4.36 (*m*, 1H, CHCONH), 3.69 (s, 3H, OCH_3_), 3.07 (d, J = 6.7 Hz, 2H, CHCH_2_Ph), 1.66–1.33 (*m*, 12H, CH_2_CH(CH_3_)_2_, C(CH_3_)_3_), 1.03- 0.75 (*m*, 6H, CH(CH_3_)_2_). **^13^C NMR** (75 MHz, CDCl_3_) δ 172.76, 171.08, 159.89, 136.27, 129.36, 128.59, 126.50, 82.20, 52.18, 50.73, 41.84, 37.88, 28.22, 24.66, 22.63, 21.88.

***N-Z-L-Ala-L-Ala-OMe (1c)***

**^1^H NMR** (300 MHz, CDCl3) δ 7.40–7.20 (*m*, 5H, ArH), 6.78 (d, J = 6.2 Hz, 1H, CONH), 5.54 (d, J = 7.5 Hz, 1H, OCONH), 5.10 (s, 2H, CH_2_Ph), 4.55 (*m*, 1H, CHCOOMe), 4.30 (*m*, 1H, CHCH_3_), 3.73 (s, 3H, OCH_3_), 1.38 (d, J = 7.0 Hz, 6H, CH_3_). **^13^C NMR** (75 MHz, CDCl3) δ 173.12, 171.86, 155.88, 136.26, 128.50, 128.14, 128.01, 66.99, 52.39, 50.36, 48.07, 18.61, 18.17. **GC-MS (EI)** *m*/*z* (% rel.): 308 (2), 249 (2), 206 (3), 178 (9), 134 (16), 102 (9), 91 (100), 88 (13), 70 (6), 59 (2). 

Analytical data matched those previously reported in the literature [54].

***N-Z-Gly-L-Val-OMe (2c)***

**^1^H NMR** (300 MHz, CDCl_3_) δ 7.42–7.26 (*m*, 5H, ArH), 6.97 (d, *J* = 8.8 Hz, 1H, CONH), 5.89 (*m*, 1H, OCONH), 5.12 (s, 2H, CH_2_Z), 4.55 (dd, *J* = 8.8, 5.2 Hz, 1H, CHCOOMe), 3.94 (d, *J* = 5.5 Hz, 2H, CH_2_CONH), 3.71 (s, 3H, OCH_3_), 2.21–2.09 (*m*, 1H, CH(CH_3_)_2_), 1.03–0.77 (*m*, 6H, CH(CH_3_)_2_). **^13^C NMR** (75 MHz, CDCl_3_) δ 172.44, 169.29, 156.73, 136.22, 128.49, 128.12, 127.99, 67.10, 57.10, 52.25, 44.41, 31.17, 18.94, 17.74. **GC-MS (EI)** *m*/*z* (% rel.): 322 (7), 290 (3), 263 (12), 215 (6), 192 (2), 130 (10), 91 (100).

***N-Z-L-Val-L-Phe-OMe (3c)***

**^1^H NMR** (300 MHz, CDCl_3_) δ 7.51–7.00 (*m*, 10H, ArH), 6.58 (d, *J* = 7.6 Hz, 1H, CONH), 5.47 (d, *J* = 8.8 Hz, 1H, OCONH), 5.16–4.96 (*m*, 2H, CH_2_Z), 4.94–4.82 (*m*, 1H, CHCOOMe), 4.11–3.98 (*m*, 1H, CHCH(CH_3_)_2_), 3.67 (s, 3H, OCH_3_), 3.15–2.99 (*m*, 2H, CH_2_Ph), 2.02 (*m*, 1H, CH(CH_3_)_2_), 0.97–0.73 (*m*, 6H, CH(CH_3_)_2_). **GC-MS (EI)** *m*/*z* (% rel.): 412 (1), 321 (3), 234 (2), 206 (15), 162 (43), 91 (100). 

## 4. Conclusions

This study demonstrates an efficient solution-phase synthesis of dipeptides using titanium tetrachloride (TiCl_4_) as a coupling agent with microwave irradiation, providing the necessary energy for the process. TiCl_4_ effectively activates the carboxyl group, promoting its reaction with the amino group of the coupling amino acid.

The reaction conditions proved highly compatible with various protecting groups, including Fmoc, Z, and Boc, which are removable under different conditions. This highlights the method’s versatility and robustness in managing the reactive functionalities of diverse amino acids.

Throughout the synthesis, the stereochemistry of the starting materials was pre-served, with no significant racemization detected by NMR analysis.

The introduction of a microwave reactor marked a significant advancement in the process. Microwave irradiation drastically reduced reaction times, enabling much faster synthesis compared to conventional heating methods. 

Additionally, this approach allowed for a lower molar ratio of TiCl_4_ to the *N*-protected amino acid, enhancing the overall efficiency and reducing energy consumption.

This method shows promise for broader applications, particularly in the synthesis of bioactive molecules, offering a more efficient and sustainable pathway for peptide synthesis.

## Figures and Tables

**Table 1:** Optimization of the microwave power in the synthesis of the model system **1a**.

Dipeptide	Temperature °C	Power (Watt)	Time (min)	Yield ^a^ (%)
**1a**	40 °C	50	60	53
**1a**	40 °C	100	40	70
**1a**	40 °C	250	20	90

^a^ isolated yield.

**Table 2:** Results of the reaction reported in Scheme 2.

Dipeptide	R_1_	R_2_	Time (min)	Yield ^a^ (%)
**2a**	H	CH_3_	30	88
**3a**	CH_2_C_6_H_4_OtBu	H	25	78
**4a**	CH_2_CH(CH_3_)_2_	H	20	80
**5a**	CH_2_CH(CH_3_)_2_	CH(CH_3_)CH_2_CH_3_	40	70
**6a**	CH_2_SCH_2_C_6_H_5_	CH_3_	35	70

^a^ isolated yield.

**Table 3:** Results of the reaction reported in Scheme 3.

Dipeptide	R_1_	R_2_	Time (min)	Yield ^a^ (%)
**1b**	CH_2_C_6_H_5_ (L)	CH_3_ (L)	20	94
**2b**	CH_2_C_6_H_5_ (L)	CH_3_ (D)	20	76
**3b**	CH_2_C_6_H_5_ (D)	CH_3_ (D)	25	71
**4b**	CH(CH_3_)CH_2_CH_3_	CH_2_CH(CH_3_)_2_	35	84
**5b**	CH_2_CH(CH_3_)_2_	CH(CH_3_)_2_	30	70
**6b**	(CH_2_)_3_NHCN(Z)NH(Z)	CH_3_	40	83
**7b**	CH_2_C_6_H_5_	CH_2_CH(CH_3_)_2_	25	89

^a^ isolated yield.

**Table 4:** Results of the reaction reported in Scheme 4.

Dipeptide	R_1_	R_2_	Time (min)	Yield ^a^ (%)
**1c**	CH_2_C_6_H_5_	CH_3_	30	81
**2c**	H	CH(CH_3_)_2_	30	75
**3c**	CH(CH_3_)_2_	CH_2_C_6_H_5_	35	65

^a^ isolated yield.

## Data Availability

Data are contained within the article and Supplementary Materials.

## Supplementary Materials

The following supporting information can be downloaded at: https://www.mdpi.com/article/10.3390/ijms25179729/s1, Compound characterization—Pages 2–32: ^1^H NMR, ^13^C NMR, MS(EI) spectra of synthesized compounds.
