# Supplementary material for: Efficient Solution-Phase Dipeptide Synthesis Using Titanium Tetrachloride and Microwave Heating
Source: Int J Mol Sci. 2024 Sep 8;25(17):9729. doi: 10.3390/ijms25179729 (PMC11395851; doi:10.3390/ijms25179729)
Supplement: Supplementary file 1 [file ijms-25-09729-s001.zip › ijms-3163289-supplementary.pdf]

Supporting Information

Efficient Solution-Phase Dipeptide Synthesis Using Titanium Tetrachloride and Microwave Heating

Palmira Alessia Cavallaro, Marzia De Santo, Rocco Marinaro, Emilia Lucia Belsito, Angelo Liguori and Antonella Leggio*

Department of Pharmacy, Health and Nutritional Sciences, University of Calabria, Via P. Bucci, 87036 Arcavacata di Rende, Italy; ; alessia.cavallaro@unical.it (P.A.C.); marzia.desanto@unical.it (M.D.S.); rocco.marinaro@unical.it (R.M.); emili-alucia.belsito@unical.it (E.L.B.); angelo.liguori@unical.it (A.L.)

Correspondence: antonella.leggio@unical.it

Table of contents

^{1}H-NMR and ^{13}C-NMR spectra of *N*-Fmoc-Dipeptides **1a-6a** Pages: 2-12

^{1}H-NMR, ^{13}C-NMR and MS (EI) spectra of *N*-Boc-Dipeptides **1b-7b** Pages: 13-26

^{1}H-NMR, ^{13}C-NMR and MS (EI) spectra of *N*-Z-Dipeptides **1c-3c** Pages: 27-32

N-Fmoc-L-Phe-L-Ala-OMe (1a)

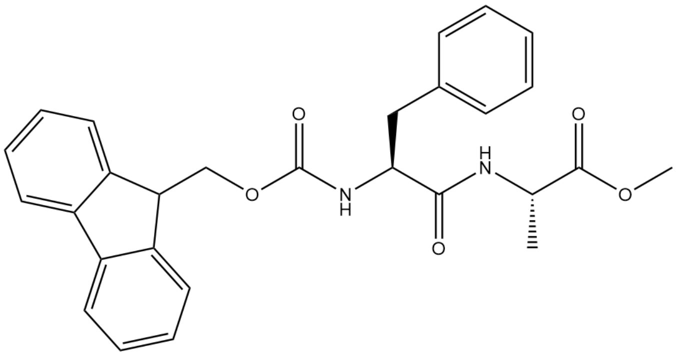

¹H NMR

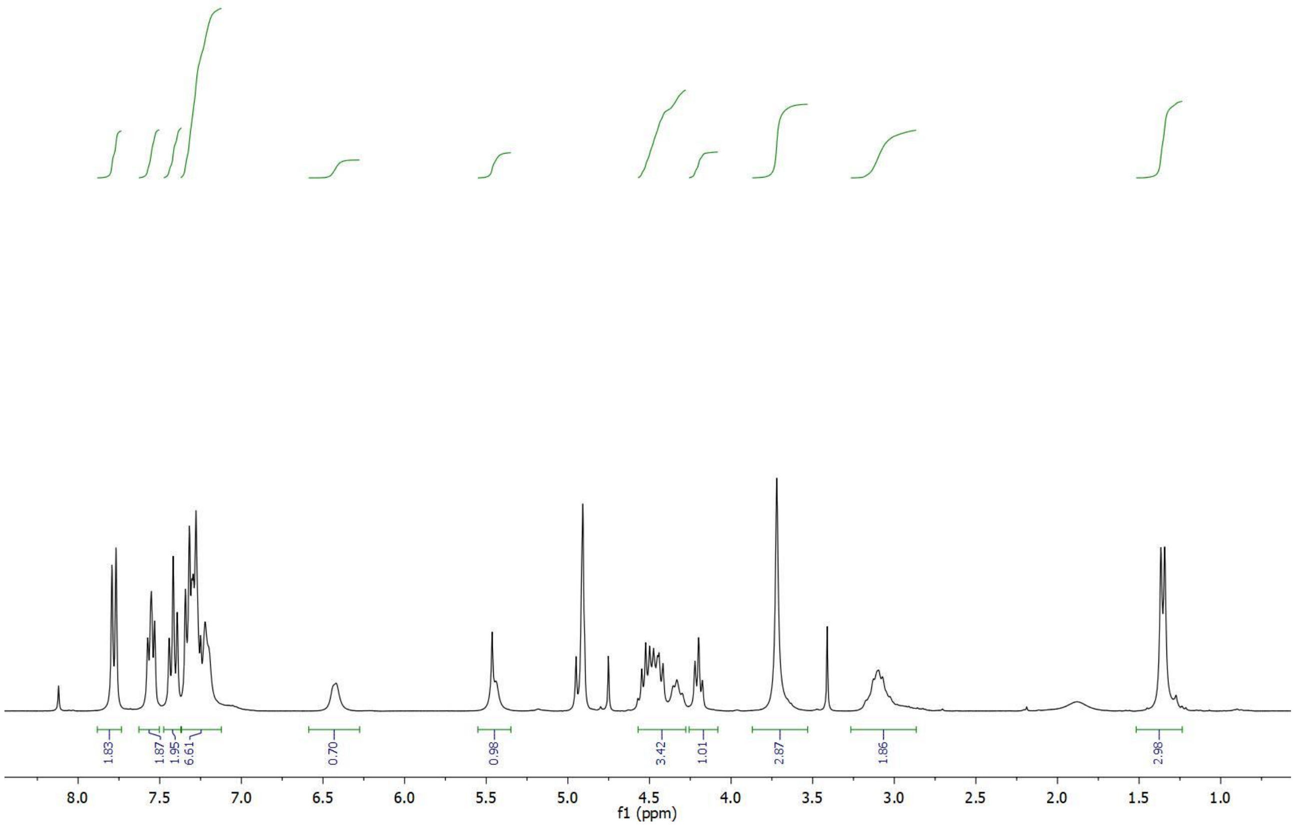

¹H NMR (300 MHz, $CDCl_3$) δ 7.78 (d, *J* = 7.5 Hz, 2H, ArH), 7.55 (t, *J* = 6.4 Hz, 2H, ArH), 7.42 (t, *J* = 7.4 Hz, 2H, ArH), 7.37 – 7.09 (m, 5H, ArH), 6.43 (s_{broad}, 1H, CONH), 5.45 (s_{broad}, 1H, OCONH), 4.66 – 4.41 (m, 3H, CH_2Fmoc, C<u>H</u>COOMe), 4.33 (m, 1H, C<u>H</u>CONH), 4.20 (t, *J* = 6.9 Hz, 1H, CHFmoc), 3.72 (s, 3H, OCH_3), 3.20-3.96 (m, 2H, C<u>H₂</u>Ph), 1.35 (d, *J* = 7.1 Hz, 3H, CH_3).

^{13}C NMR

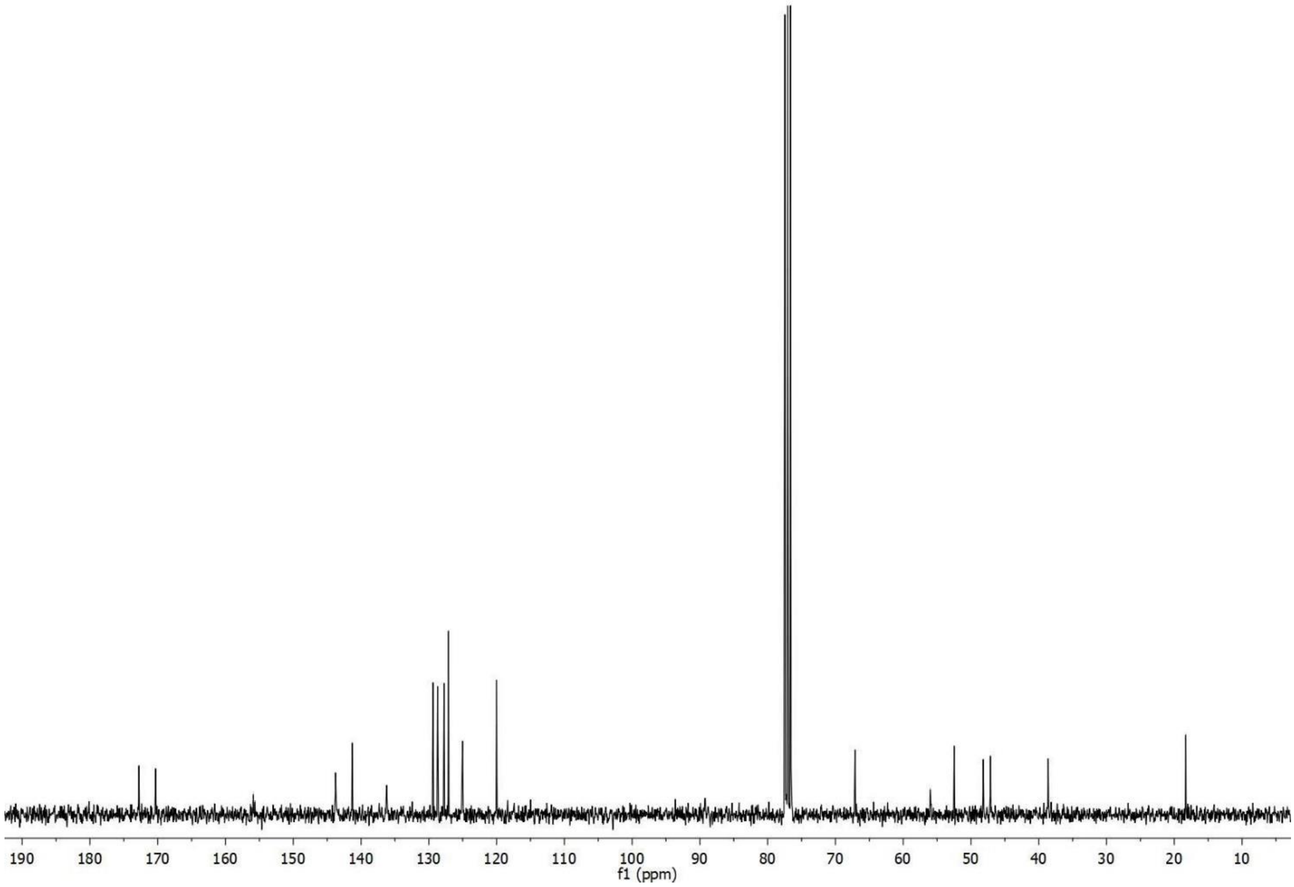
190
180
170
160
150
140
130
120
110
100
90
80
70
60
50
40
30
20
10
f1 (ppm)

N-Fmoc-Gly-L-Ala-OMe (2a)

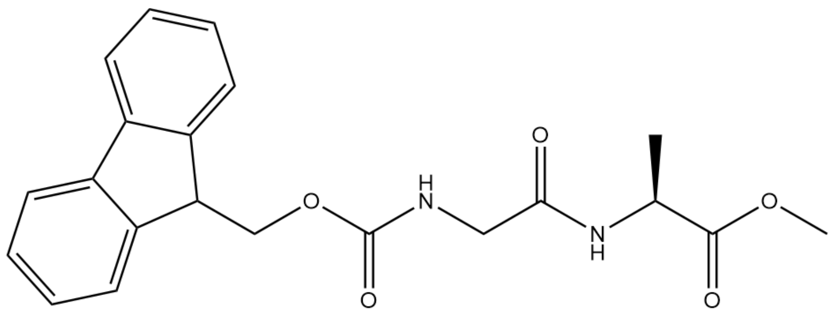

¹H NMR

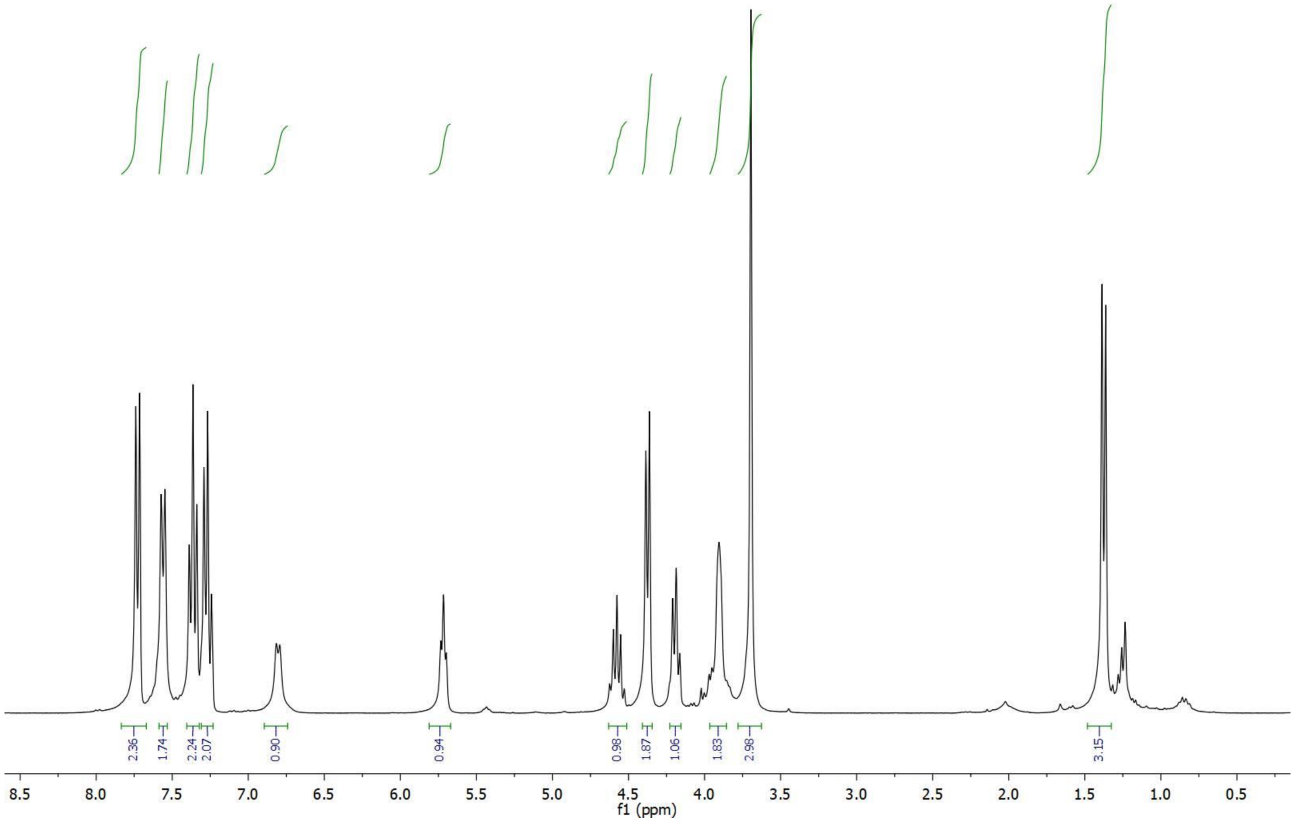

¹H NMR (300 MHz, $CDCl_3$) δ 7.73 (d, J = 7.5 Hz, 2H, ArH), 7.56 (d, J = 7.3 Hz, 2H, ArH), 7.47 - 7.19 (m, 4H, ArH), 6.80 (d_{broad}, J = 6.4 Hz, 1H, CONH), 5.72 (t_{broad}, J = 5.4 Hz, 1H, OCONH), 4.78 - 4.48 (m, 1H, $C\underline{H}COOCH_3$), 4.37 (d, J = 7.0 Hz, 2H, CH_2Fmoc), 4.19 (t, J = 7.0 Hz, 1H, CHFmoc), 4.05 -3.81 (m, 2H, $C\underline{H_2}CONH$), 3.69 (s, 3H, OCH_3), 1.37 (d, J = 7.1 Hz, 3H, $C\underline{H_3}CH$).

^{13}C NMR

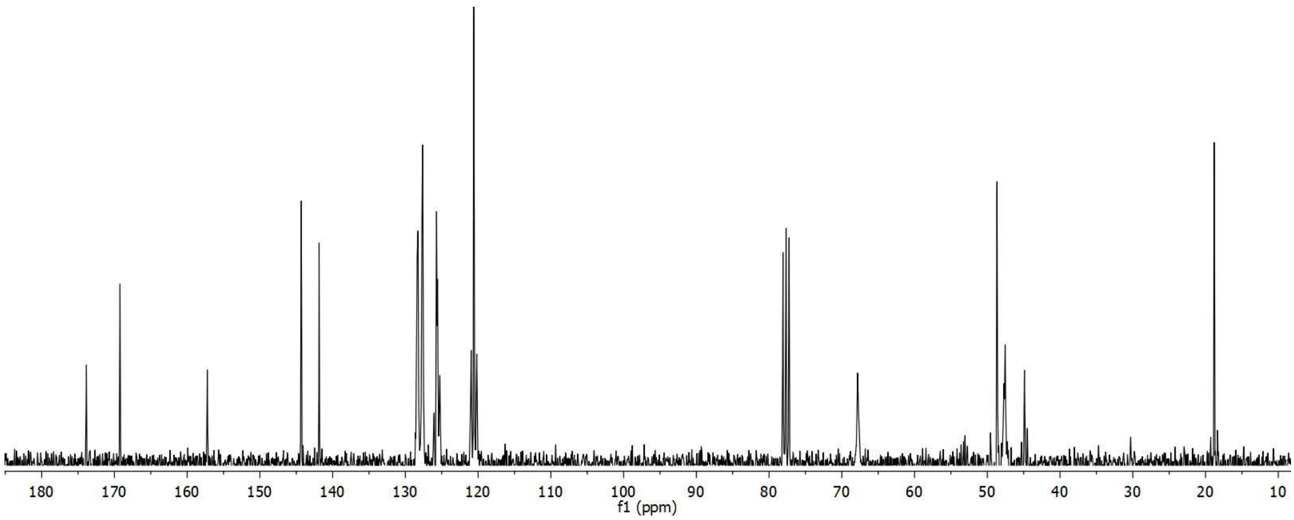

180 170 160 150 140 130 120 110 100 90 80 70 60 50 40 30 20 10
f1 (ppm)

N-Fmoc-L-Tyr(tBu)-Gly-OMe (3a)

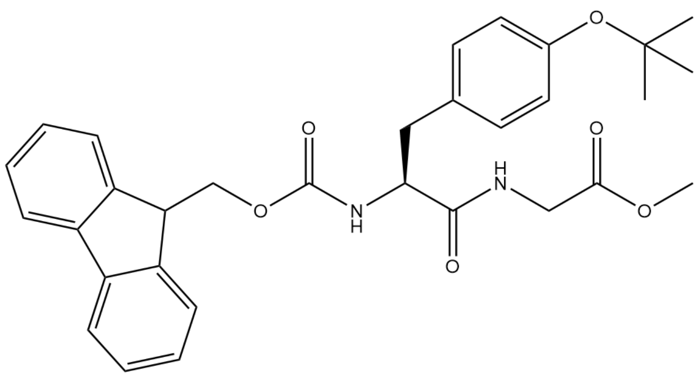

^{1}H NMR

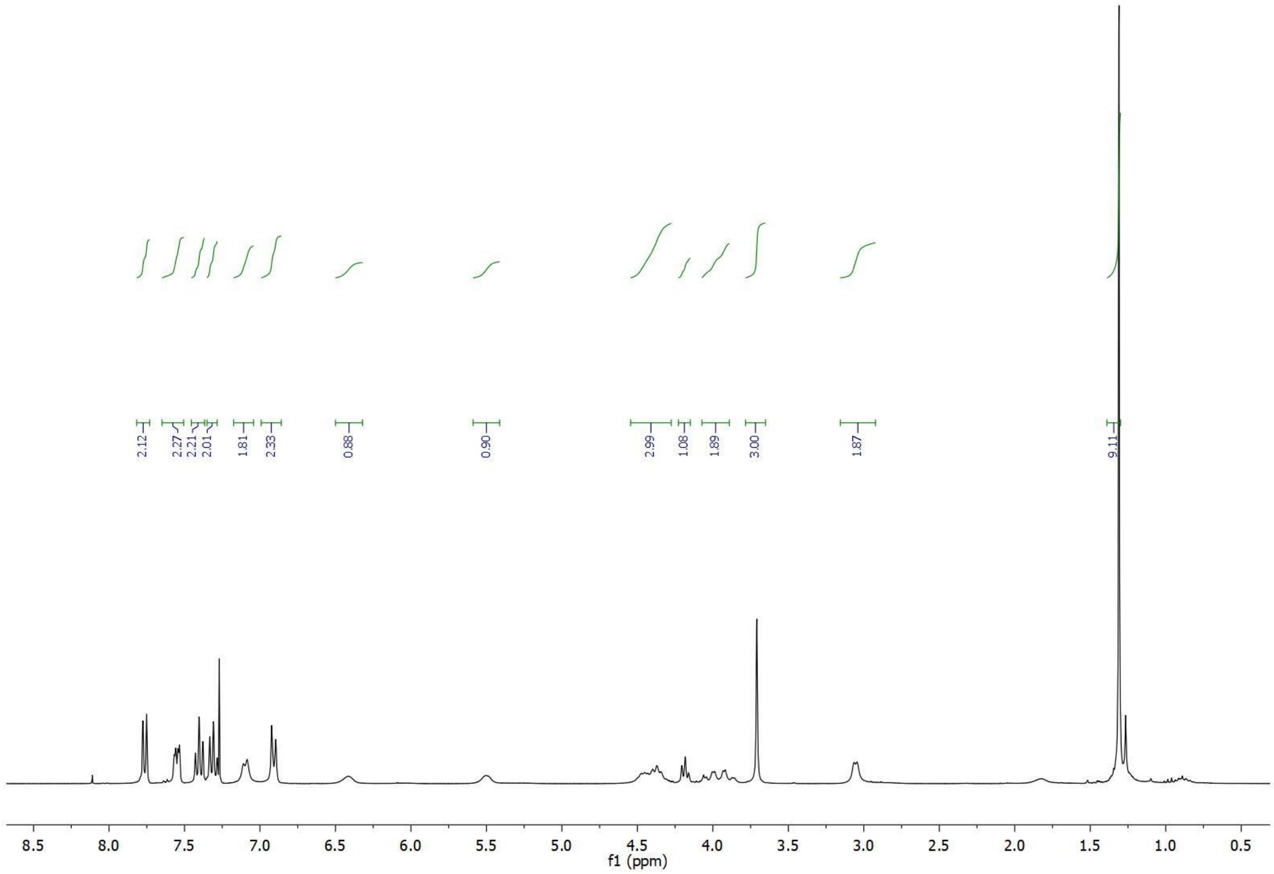

^{1}H NMR (300 MHz, $CDCl_3$) 7.76 (d, J = 7.4 Hz, 2H, ArH), 7.59-7.49 (m, 2H, ArH), 7.40 (t, J = 7.2 Hz, 2H, ArH), 7.32 (d, J = 7.2 Hz, 2H, ArH), 7.17-7.03 (m, 2H, ArH), 6.91 (d, J = 7.4 Hz, 2H, ArH), 6.41 (s_{broad}, 1H, OCONH), 5.50 (s_{broad}, 1H, CONH), 4.55 – 4.28 (m, 3H, CH_2Fmoc, CHCONH), 4.18 (t, *J* = 6.8 Hz, 1H, CHFmoc), 4.07 – 3.89 (m, 2H, CH_2COOMe), 3.71 (s, 3H, OCH_3), 3.11-2.99 (m 2H, CH_2-Tyr), 1.31 (s, 9H, $C(CH_3)_3$).

^{13}C NMR

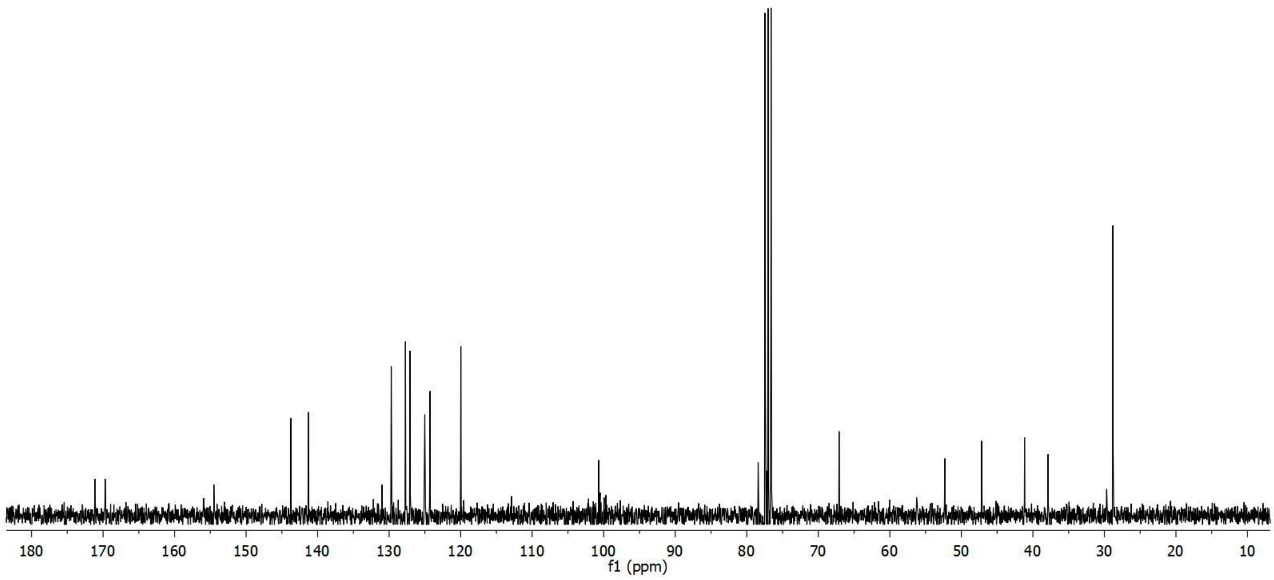
180
170
160
150
140
130
120
110
100
90
80
70
60
50
40
30
20
10
f1 (ppm)

N-Fmoc-L-Leu-Gly-OMe (4a)

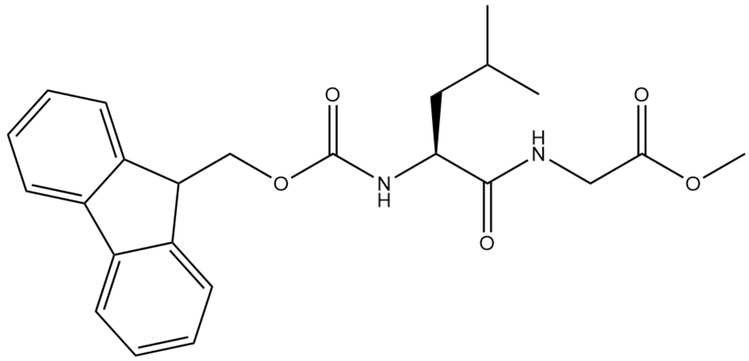

^{1}H NMR

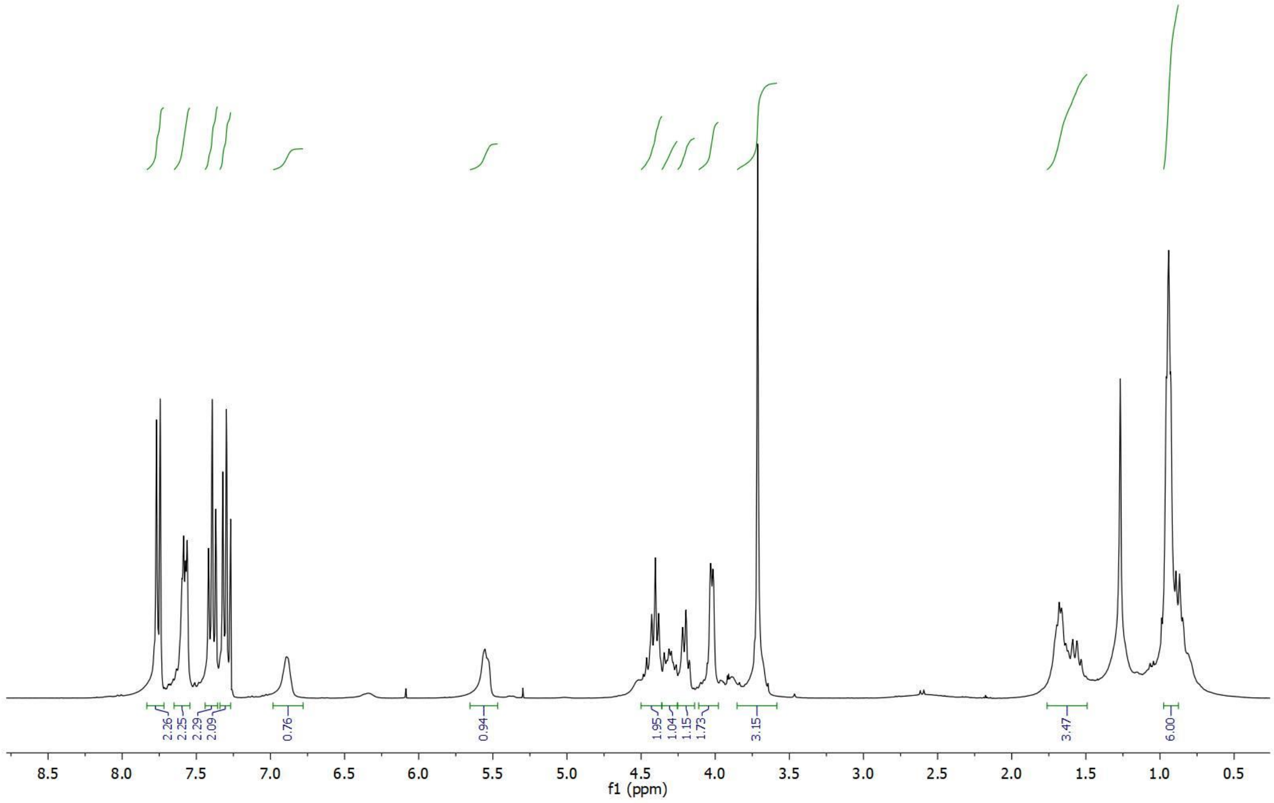

^{1}H NMR (300 MHz, $CDCl_3$) δ 7.76 (d, *J* = 7.5 Hz, 2H, ArH), 7.65-7.53 (m, 2H, ArH), 7.38 (d, *J* = 7.4 Hz, 2H, ArH), 7.35 – 7.27 (m, 2H, ArH), 6.89 (s_{broad}, 1H, CONH), 5.55 (s_{broad}, 1H, OCONH), 4.50-4.37 (m, 2H, CH_2Fmoc), 4.34–4.26 (m, 1H, C<u>H</u>CONH), 4.20 (t, J = 6.9 Hz, 1H, CHFmoc) 4.02 (m, 2H, C<u>H</u>$_2$COOCH$_3$), 3.71 (s, 3H, OCH_3), 1.80 – 1.46 (m, 3H, C<u>H</u>$_2$C<u>H</u>), 1.04 – 0.88 (m, 6H, CH(C<u>H</u>$_3$)$_2$).

^{13}C NMR

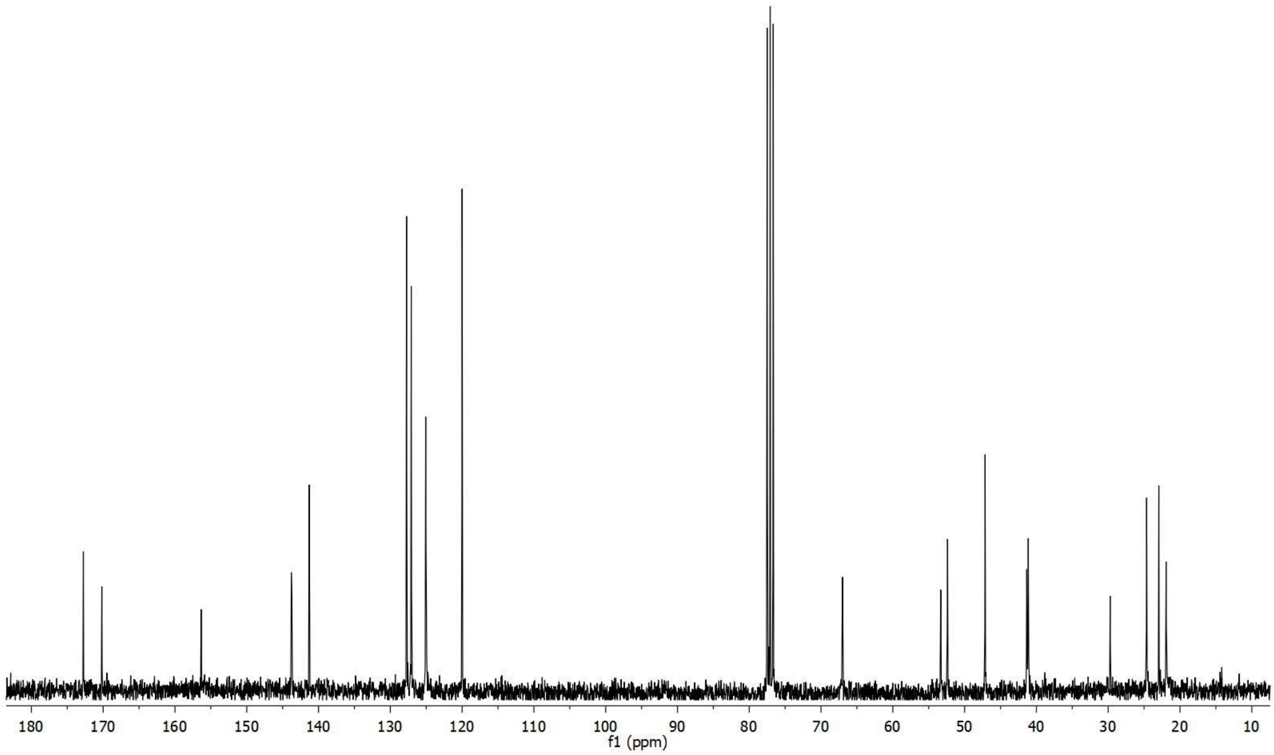

180
170
160
150
140
130
120
110
100
90
80
70
60
50
40
30
20
10
f1 (ppm)

N-Fmoc-L-Leu-L-Ile-OMe (5a)

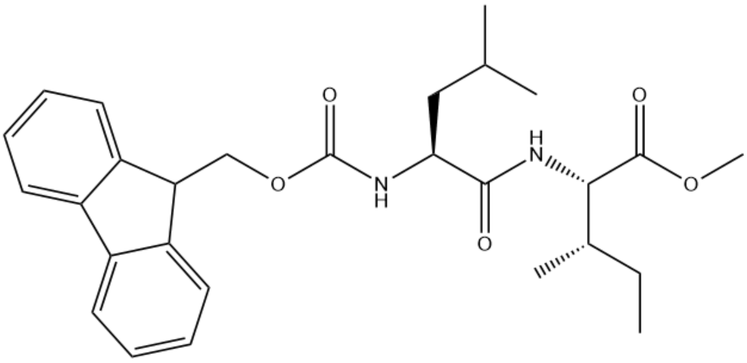

^{1}H NMR

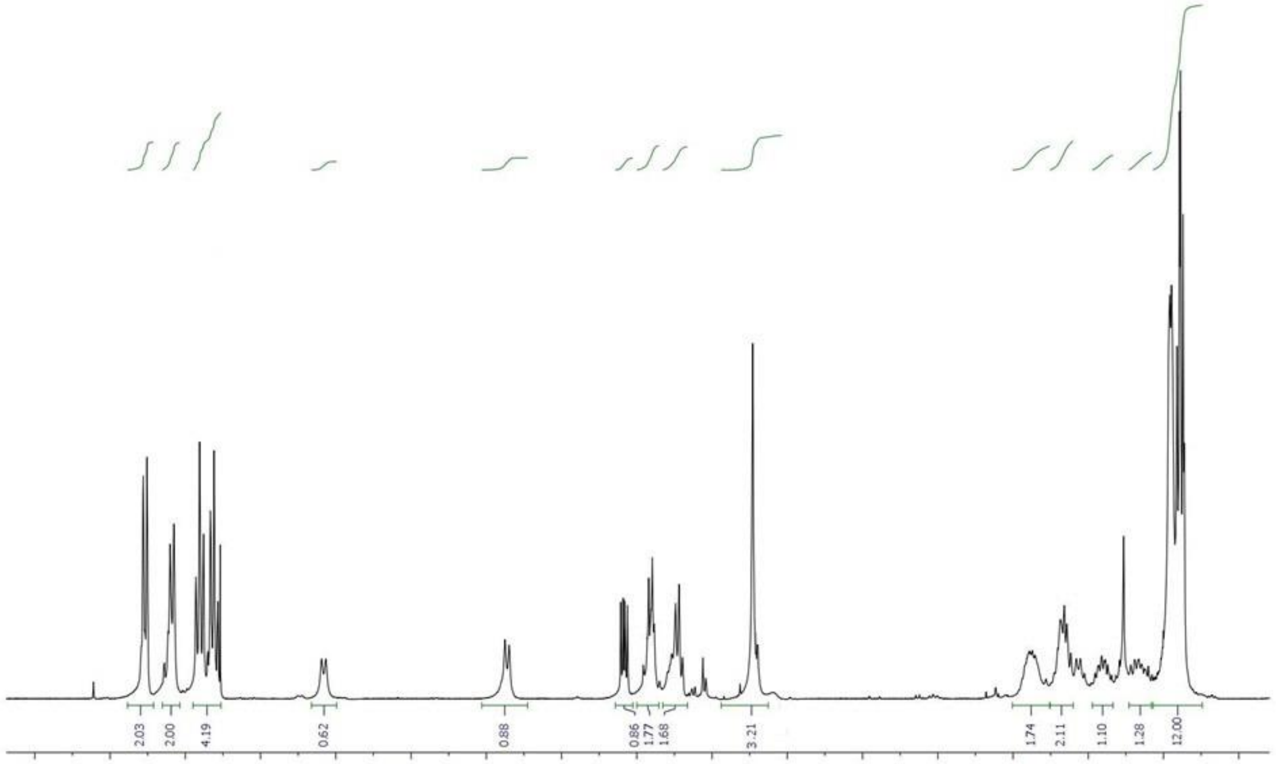

^{1}H-NMR (300 MHz, $CDCl_3$) δ 7.77 (d, J = 7.5 Hz, 2H, ArH), 7.59 (d, J = 7.5 Hz, 2H, ArH), 7.49 – 7.20 (m, 4H, ArH), 6.58 (d, J = 8.4 Hz, 1H, CONH), 5.36 (d, J = 8.4 Hz, 1H, OCONH), 4.58 (dd, J = 8.4, 5.1 Hz, 1H, C<u>H</u>COOMe), 4.50-4.35 (m, 2H, CH_2-Fmoc), 4.33-4.16 (m, 2H, CH-Fmoc, C<u>H</u>CONH), 3.73 (s, 3H, OCH_3), 1.98-1.75 (m, 2H, C<u>H</u>CH_3, C<u>H</u>$_2$CH$(CH_3)_2$), 1.74-1.59

(m, 2H, C<u>H</u>$_2$C<u>H</u>(CH$_3$)$_2$), 1.47 -1.33 (m, 1H, C<u>H</u>$_2$CH$_3$), 1.23 – 1.08 (m, 1H, C<u>H</u>$_2$CH$_3$), 1.07 – 0.74 (m, 12H, CHC<u>H</u>$_3$, CH$_2$C<u>H</u>$_3$, CH$_2$CH(C<u>H</u>$_3$)$_2$).

^{13}C NMR

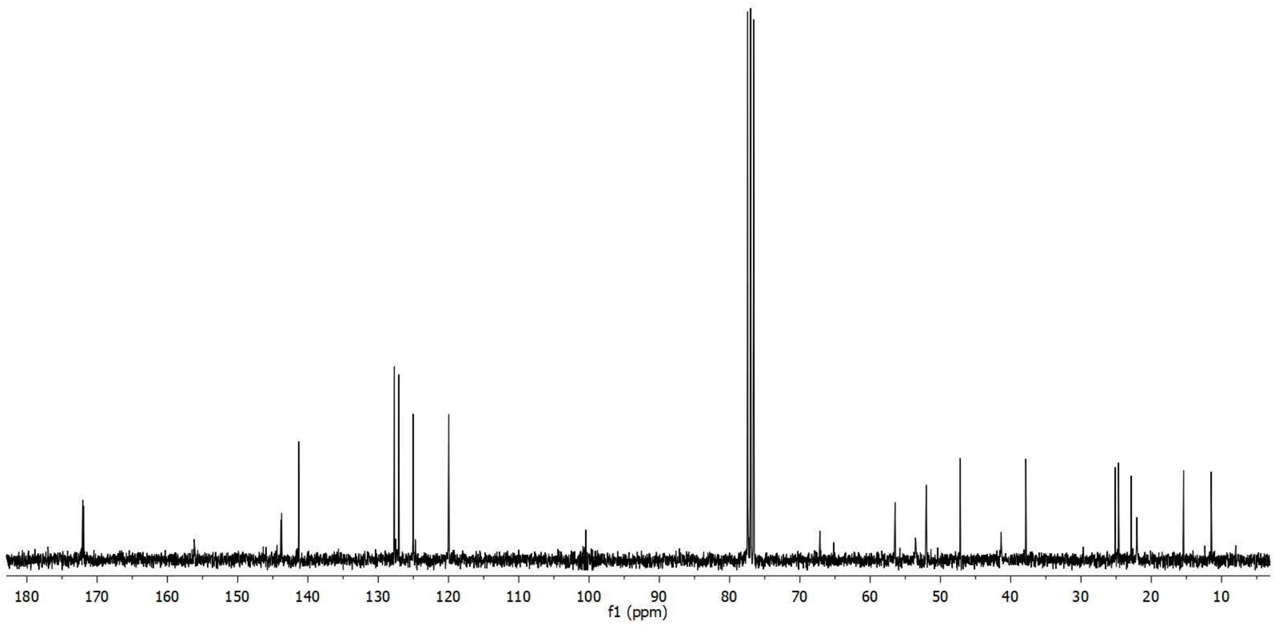

N-Fmoc-L-Cys(Bzl)-L-Ala-OMe (6a)

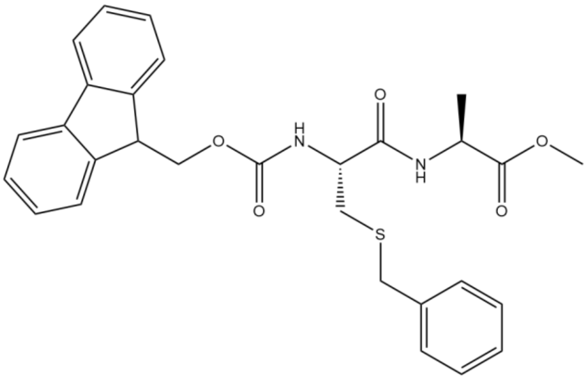

¹H NMR

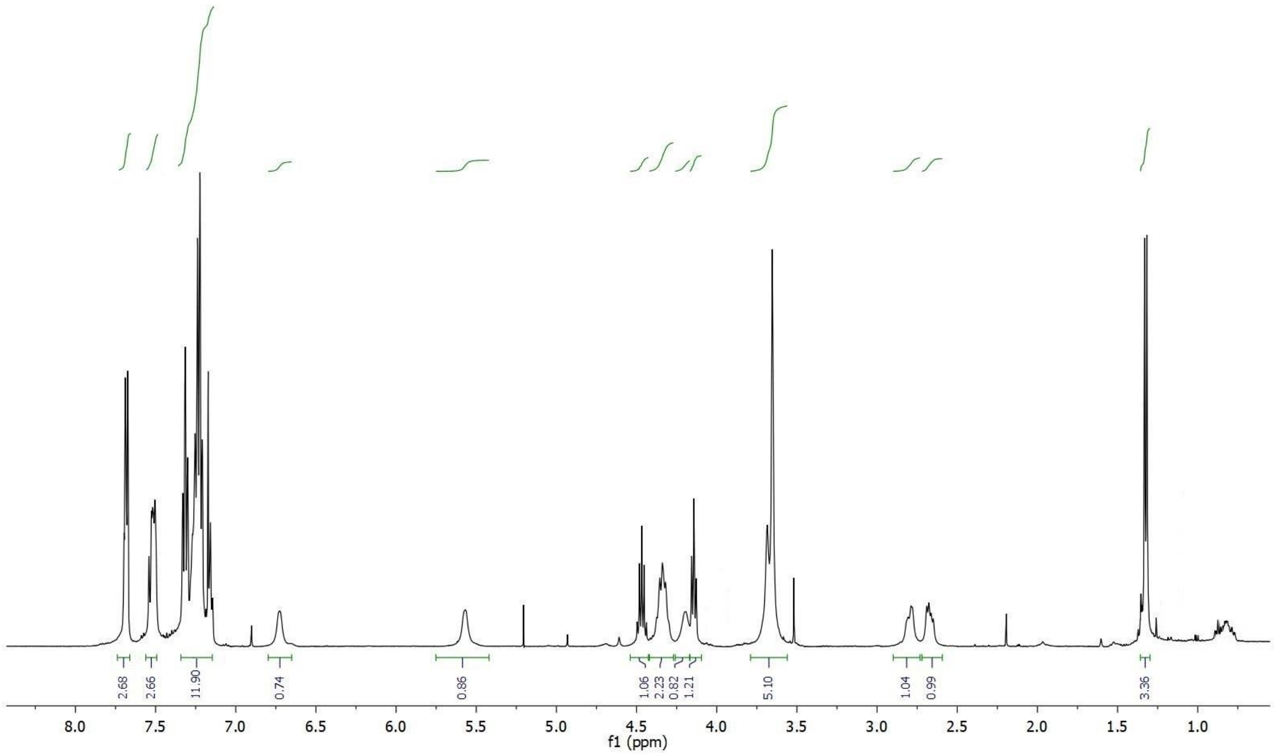

^{1}H NMR (300 MHz, $CDCl_3$) δ 7.68 (d, 2H, J = 7.5 Hz, ArH), 7.58-7.46 (m, 2H, ArH), 7.42-7.09 (m, 9H, ArH), 6.72 (s_{broad}, 1H, CONH), 5.57 (s_{broad}, 1H, OCONH), 4.47 (m, 1H, $CHCOOCH_3$), 4.41-4.26 (m, 2H, $C\underline{H}_2$-Fmoc), 4.25-4.15 (m, 1H, CHCONH), 4.14-4.10 (m, 1H, CH-Fmoc), 3.75-3.56 (m, 5H, $SC\underline{H}_2Ph$, $OC\underline{H}_3$), 2.80 (m, 1H, $CH\underline{CH_2}S$), 2.67 (m, 1H, $CH\underline{CH_2}S$), 1.32 (d, 3H, J = 7.2 Hz, CH_3).

N-Boc-L-Phe-L-Ala-OMe (1b)

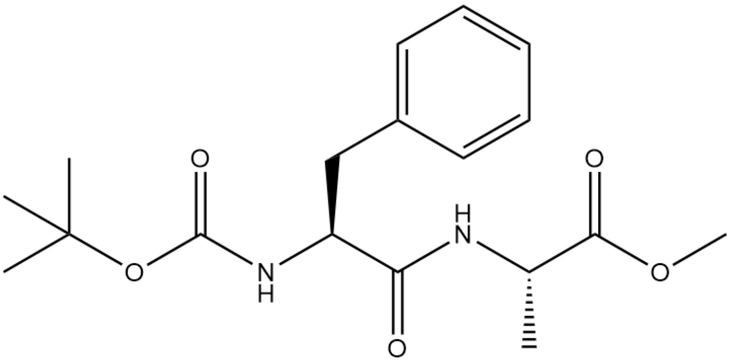

^{1}H NMR

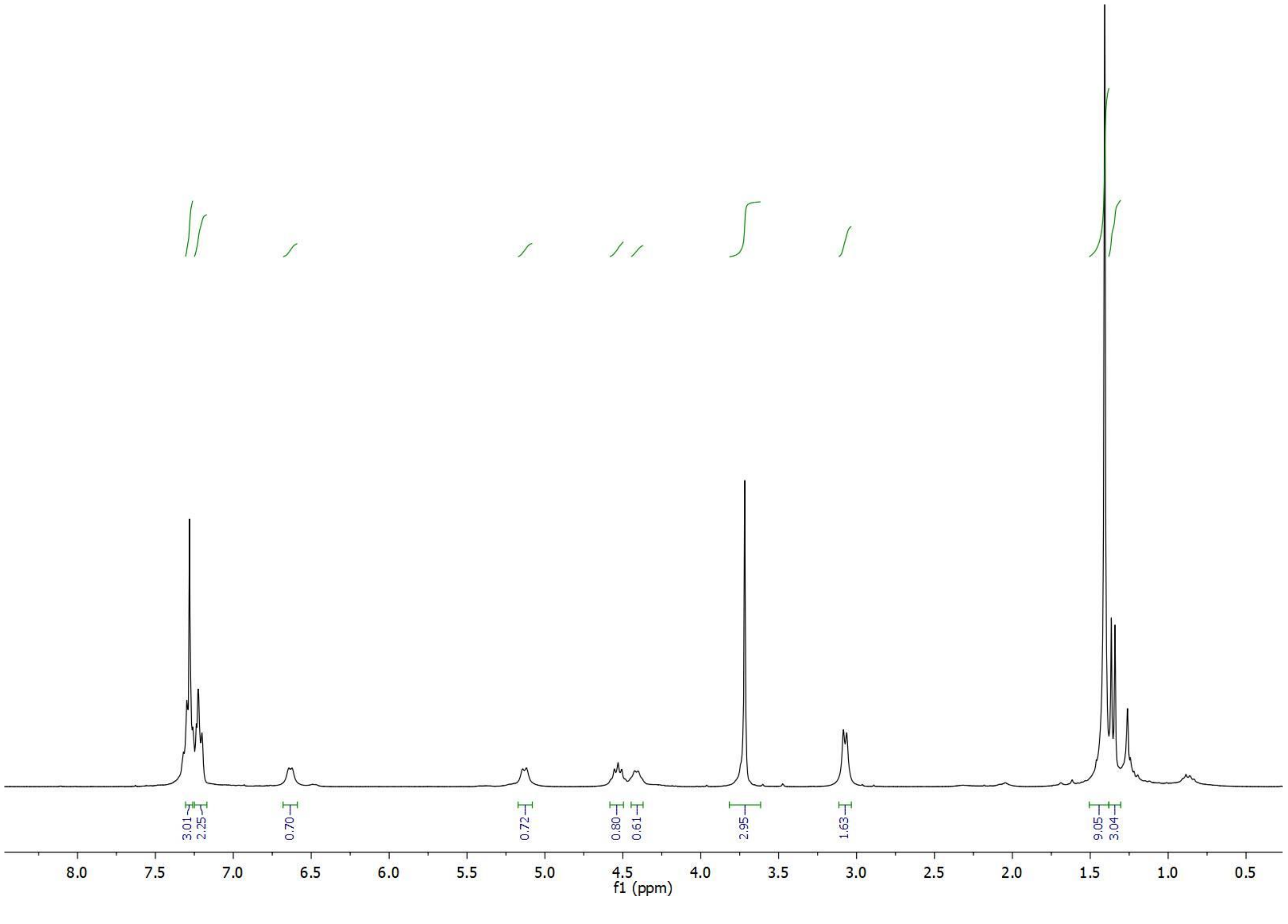

^{1}H NMR (300 MHz, $CDCl_3$) δ 7.42-7.11 (m, 5H, ArH), 6.63 (s_{broad}, 1H, CONH), 5.14 (s_{broad}, 1H, OCONH), 4.54 (m, 1H, C$\underline{H}$COOMe), 4.40 (m, 1H, C$\underline{H}$$CH_2$Ph), 3.72 (s, 3H, OCH_3), 3.07 (d, J = 6.2 Hz, 2H, CH_2Ph), 1.41 (s, 9H, $C(CH_3)_3$), 1.35 (d, J = 7.2 Hz, 3H, CHC$\underline{H}_3$).

^{13}C NMR

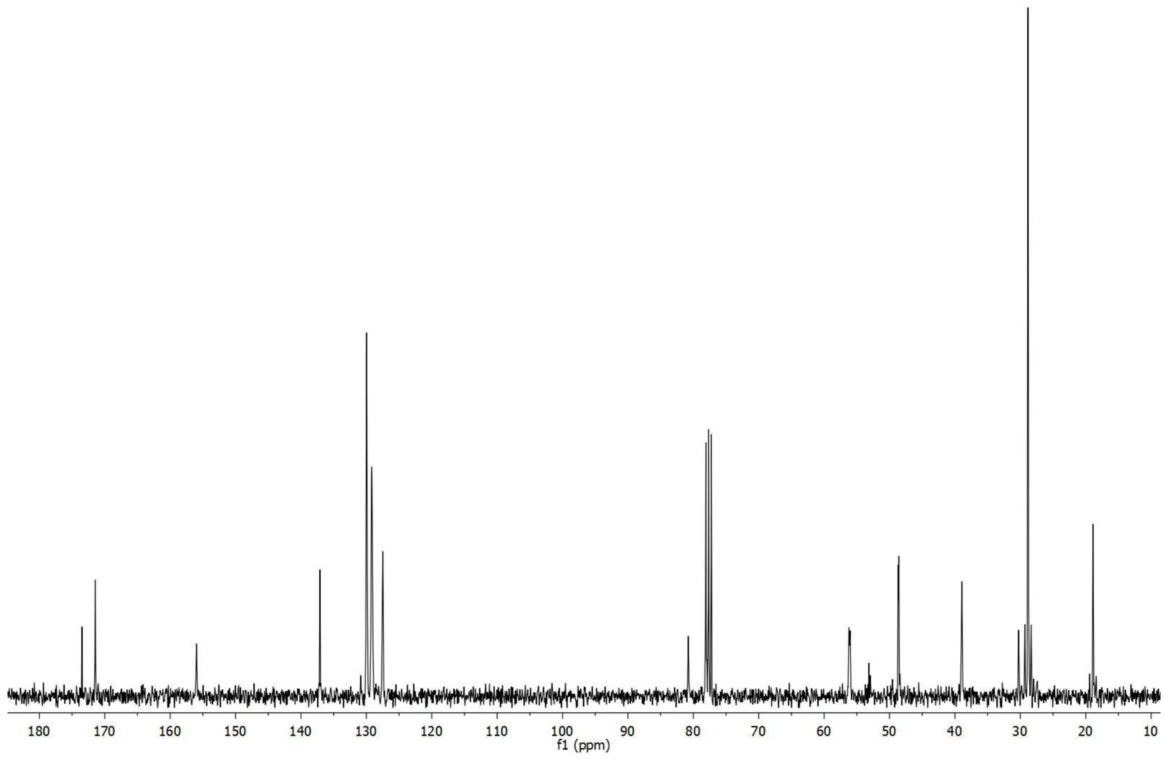
180
170
160
150
140
130
120
110
100
90
80
70
60
50
40
30
20
10
f1 (ppm)

MS (EI)

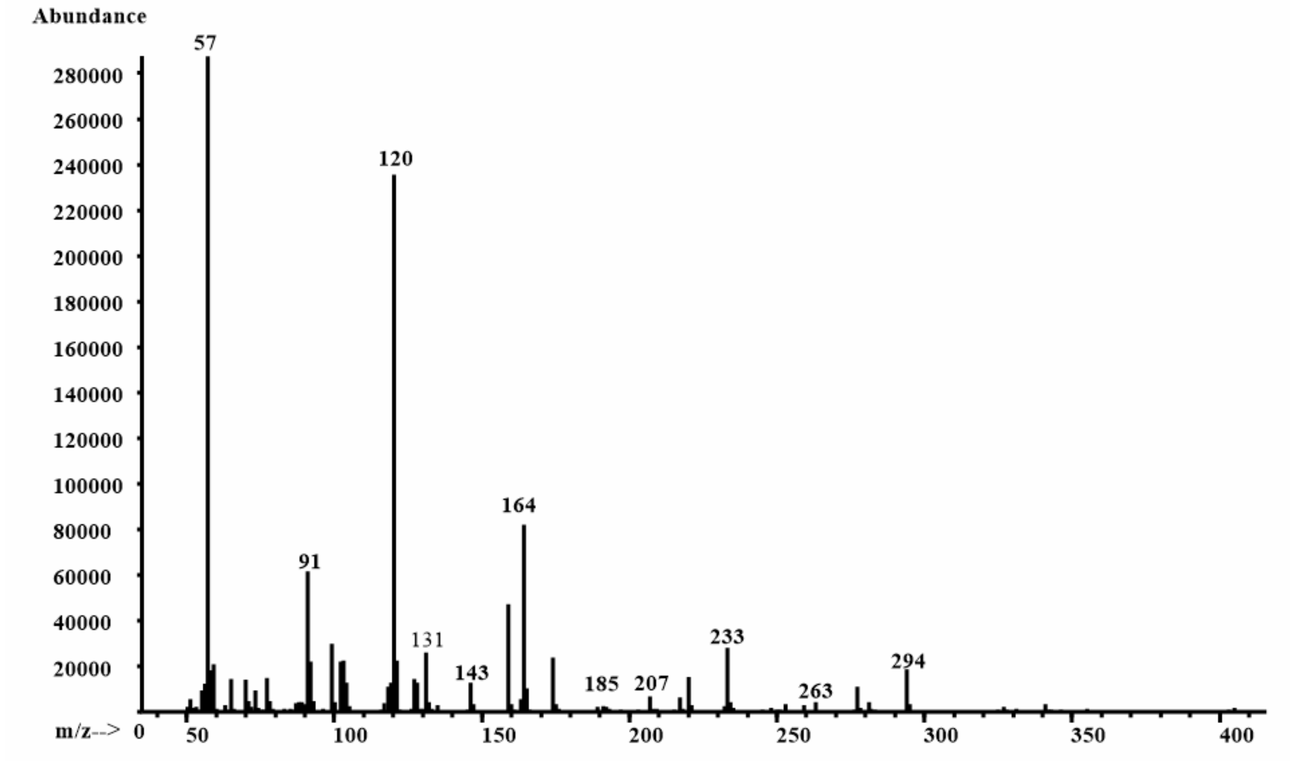
Abundance
280000
260000
240000
220000
200000
180000
160000
140000
120000
100000
80000
60000
40000
20000
57
120
91
131
143
164
185
207
233
263
294
m/z--> 0
50
100
150
200
250
300
350
400

N-Boc-L-Phe-D-Ala-OMe (2b)

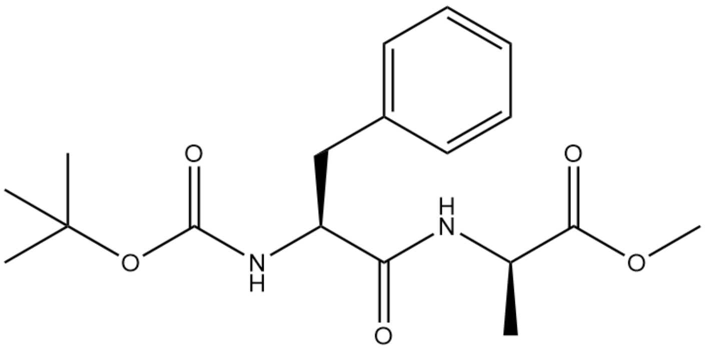

^{1}H NMR

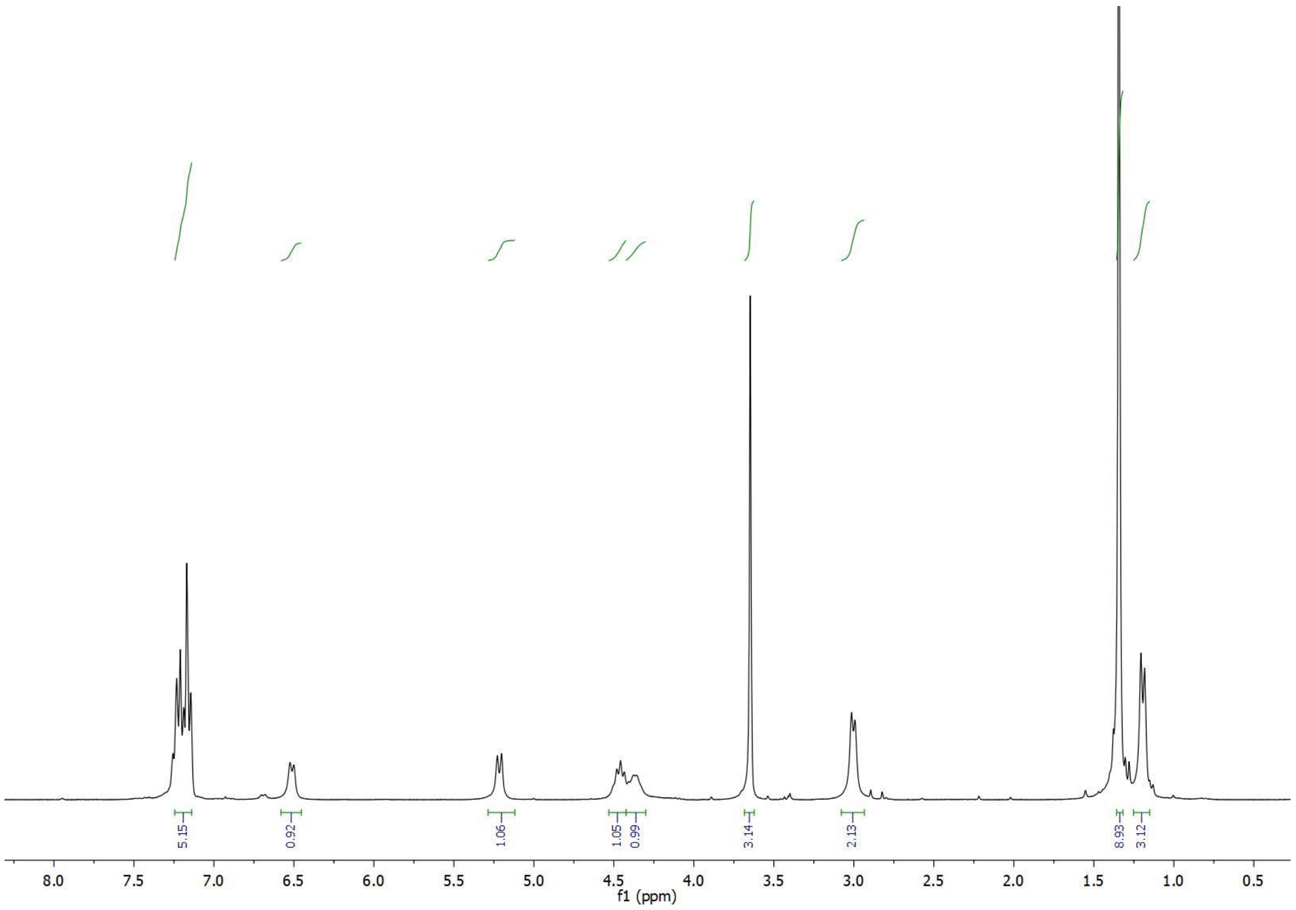

^{1}H NMR (300 MHz, $CDCl_3$) δ 7.24 – 7.14 (m, 5H, ArH), 6.51 (d, J = 7.0 Hz, 1H, CONH), 5.22 (d, J = 8.0 Hz, 1H, OCONH), 4.45 (m, 1H, C<u>H</u>COOMe), 4.35 (m, 1H, C<u>H</u>CH_2Ph), 3.65 (s, 3H, OCH_3), 3.01 (d, J = 6.5 Hz, 2H, CH_2Ph), 1.34 (s, 9H, $C(CH_3)_3$), 1.19 (d, J = 6.9 Hz, 3H, CHC<u>H</u>$_3$).

^{13}C NMR

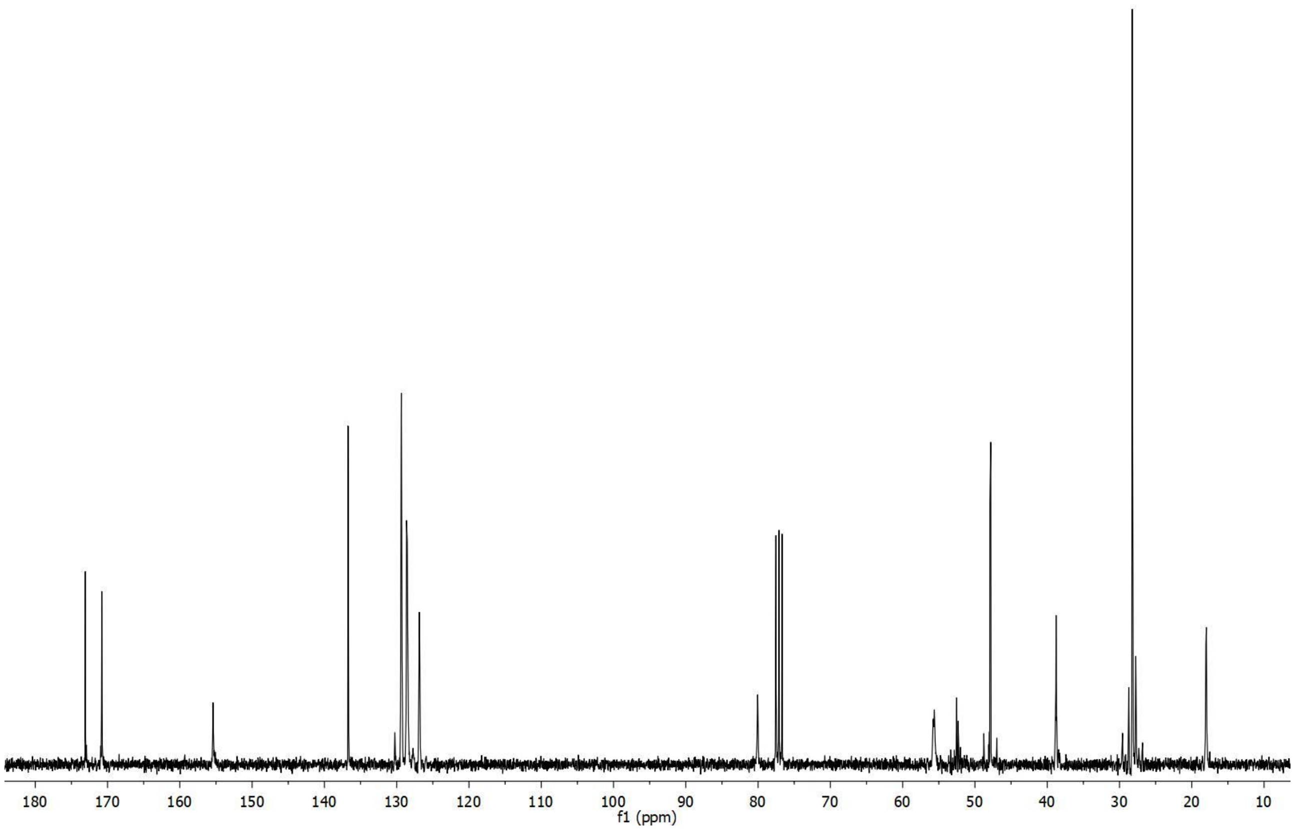

180
170
160
150
140
130
120
110
100
90
80
70
60
50
40
30
20
10
f1 (ppm)

MS (EI)

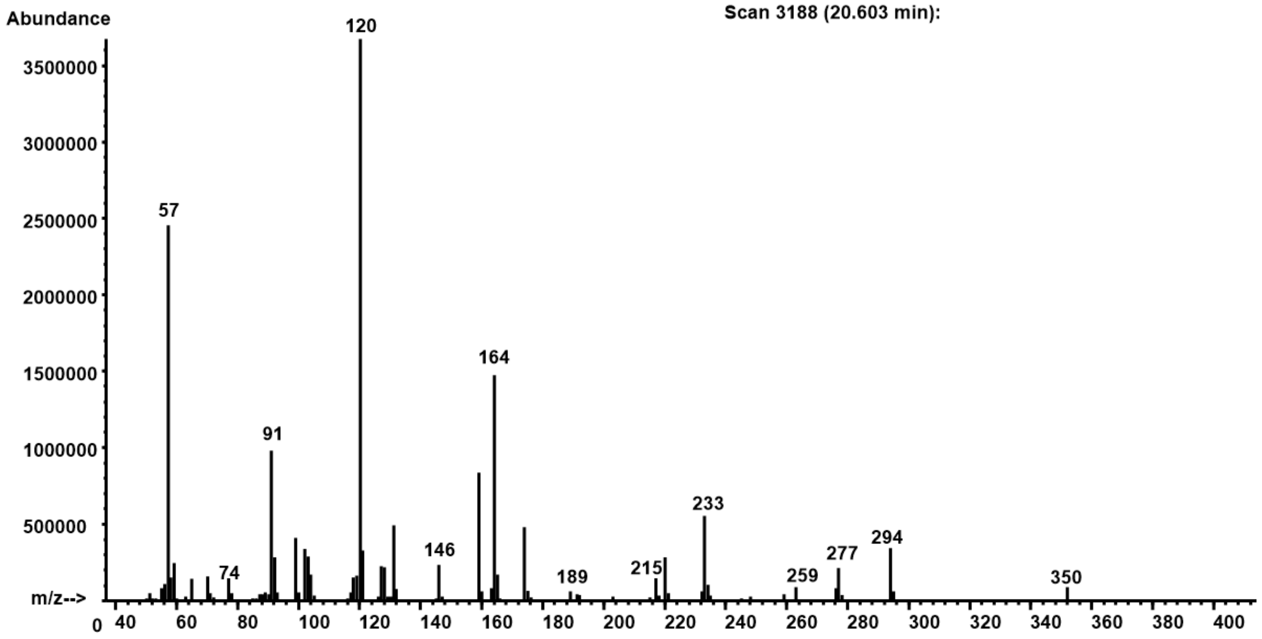

Abundance
Scan 3188 (20.603 min):
3500000
3000000
2500000
2000000
1500000
1000000
500000
m/z-->
0
40
60
80
100
120
140
160
180
200
220
240
260
280
300
320
340
360
380
400
57
74
91
120
146
164
189
215
233
259
277
294
350

_N_-Boc-D-Phe-D-Ala-OMe (3b)

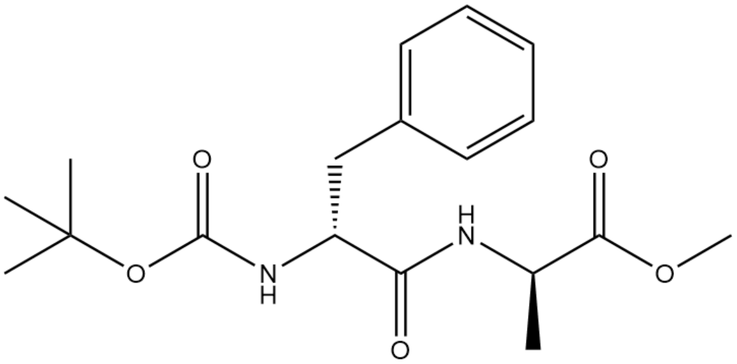

^{1}H NMR

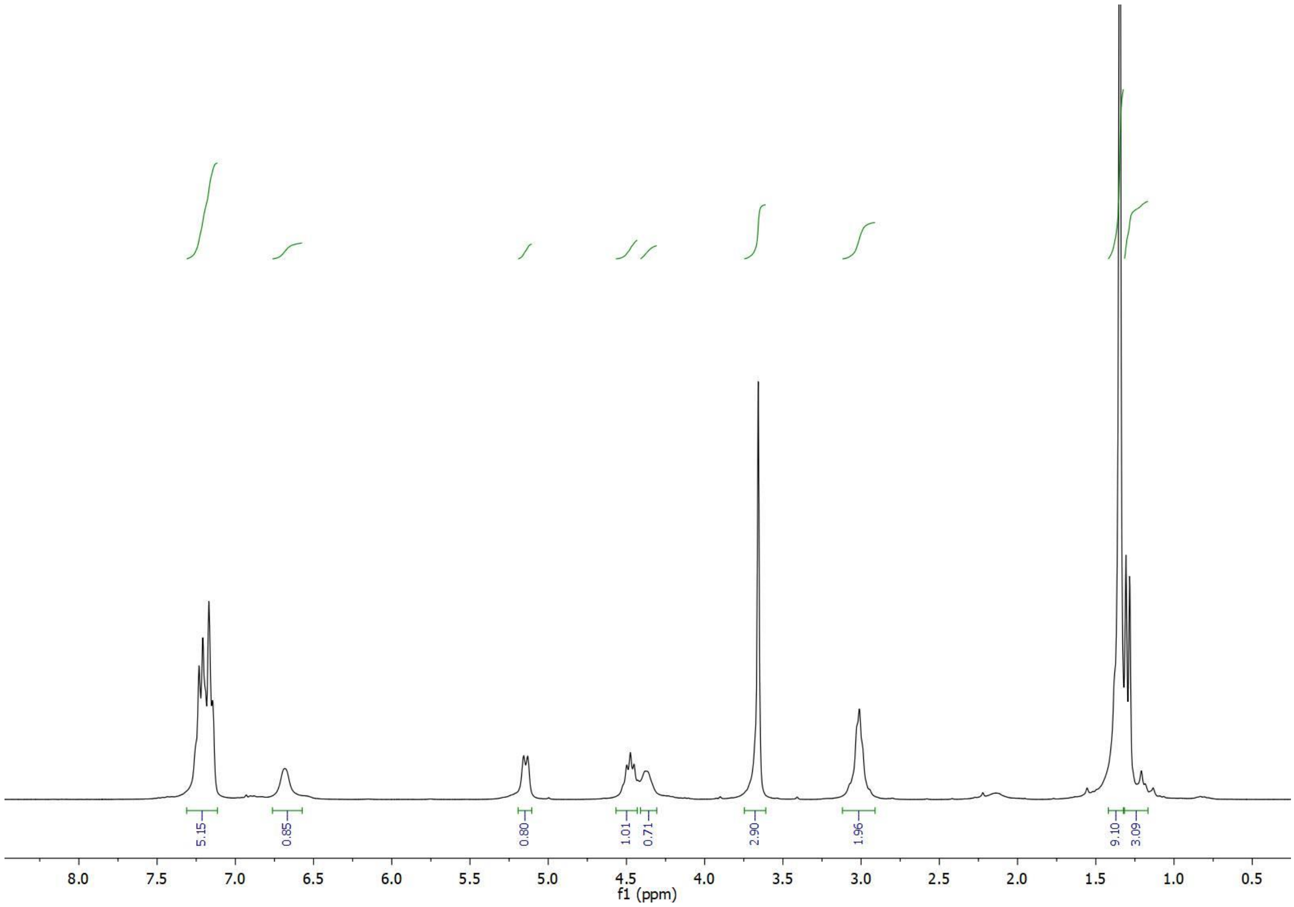

^{1}H NMR (300 MHz, $CDCl_3$) δ 7.17 (t, *J* = 9.4 Hz, 5H, ArH), 6.69 (s, 1H, CONH), 5.14 (d, *J* = 7.6 Hz, 1H, OCONH), 4.48 (t, *J* = 6.8 Hz, 1H, C<u>H</u>COOMe), 4.36 (m, 1H, C<u>H</u>CH_2Ph), 3.66 (s, 3H, OCH_3), 3.01 (s, 2H, CH_2Ph), 1.35 (s, 9H, $C(CH_3)_3$), 1.30 (d, *J* = 7.2 Hz, 3H, CHC<u>H</u>$_3$).

^{13}C NMR

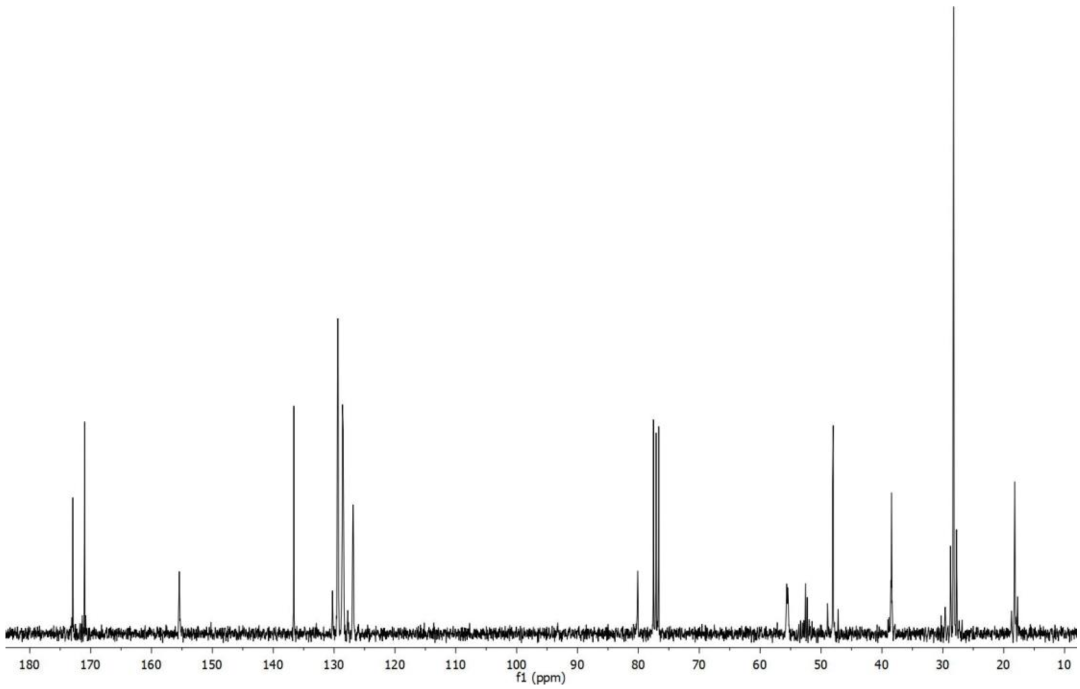
180
170
160
150
140
130
120
110
100
90
80
70
60
50
40
30
20
10
f1 (ppm)

MS (EI)

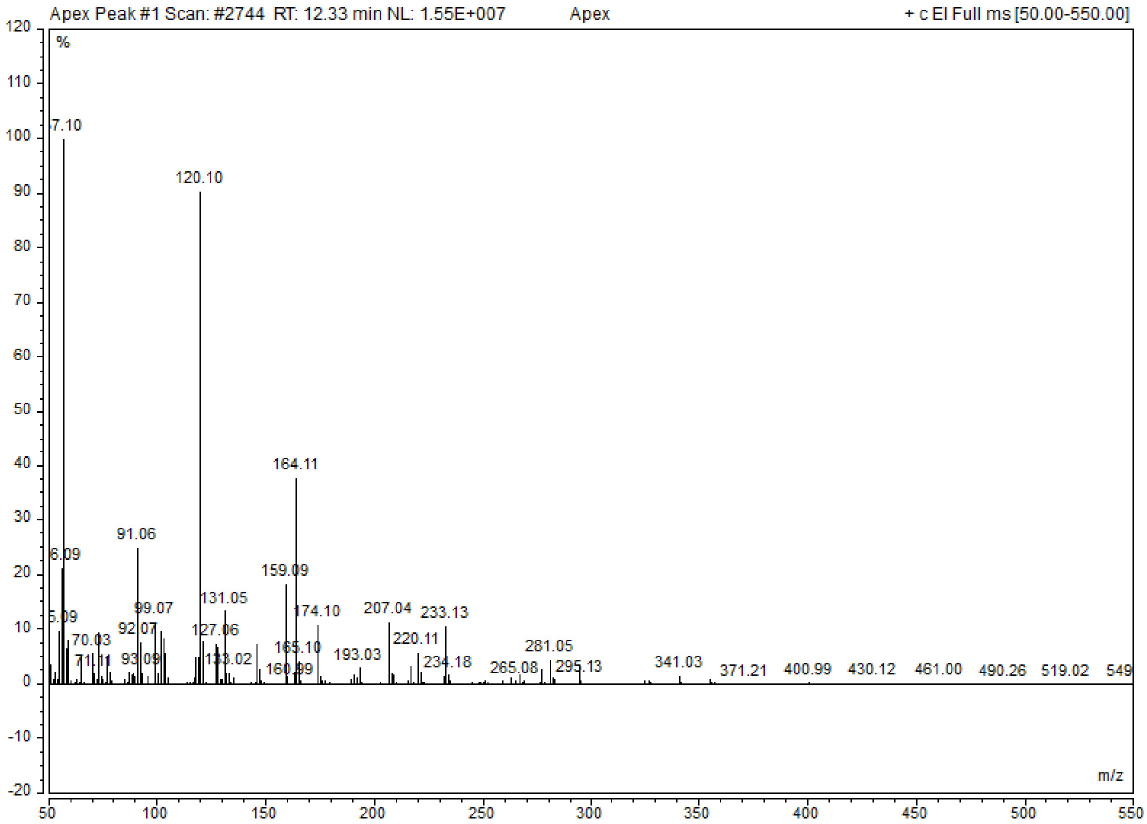
Apex Peak #1 Scan: #2744 RT: 12.33 min NL: 1.55E+007
Apex
+ c EI Full ms [50.00-550.00]
%
120.10
164.11
91.06
159.09
131.05
99.07
174.10
207.04
233.13
220.11
281.05
193.03
341.03
371.21
400.99
430.12
461.00
490.26
519.02
m/z

N-Boc-L-Ile-L-Leu-OMe (4b)

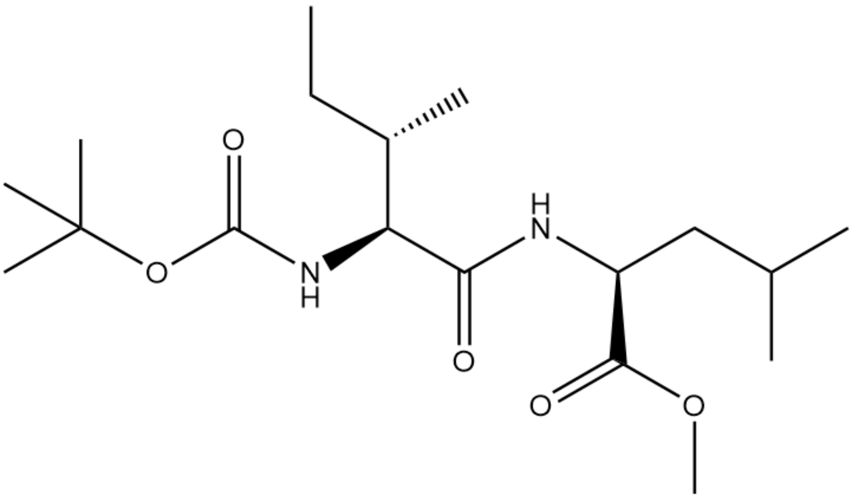

^{1}H NMR

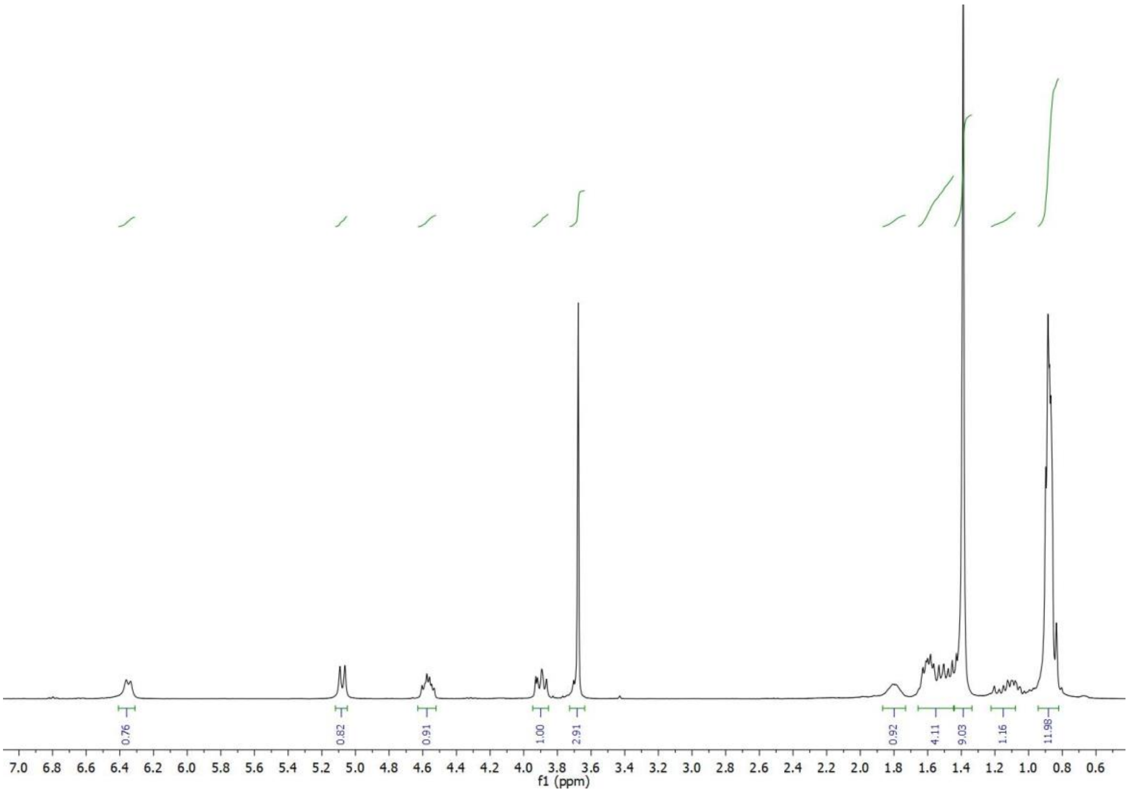

^{1}H-NMR (300 MHz, $CDCl_3$) δ 6.35 (d, *J* = 7.6 Hz, 1H, CONH), 5.08 (d, *J* = 8.9 Hz, 1H, OCONH), 4.57 (m, 1H, CHCOOMe), 3.89 (m, 1H, CHCONH), 3.68 (s, 3H, OCH_3), 1.81 (s_{broad}, 1H, $CHCH_3$), 1.66 – 1.45 (m, 4H, CH_2CH_3), CH_2CH), 1,38 (s, 9H, $C(CH_3)_3$), 1.22 – 1.08 (m, 1H, $CH(CH_3)_2$), 0.99-0.89 (m, 12H, $CH(CH_3)_2$, $CH_2(CH_3)_2$).

^{13}C NMR

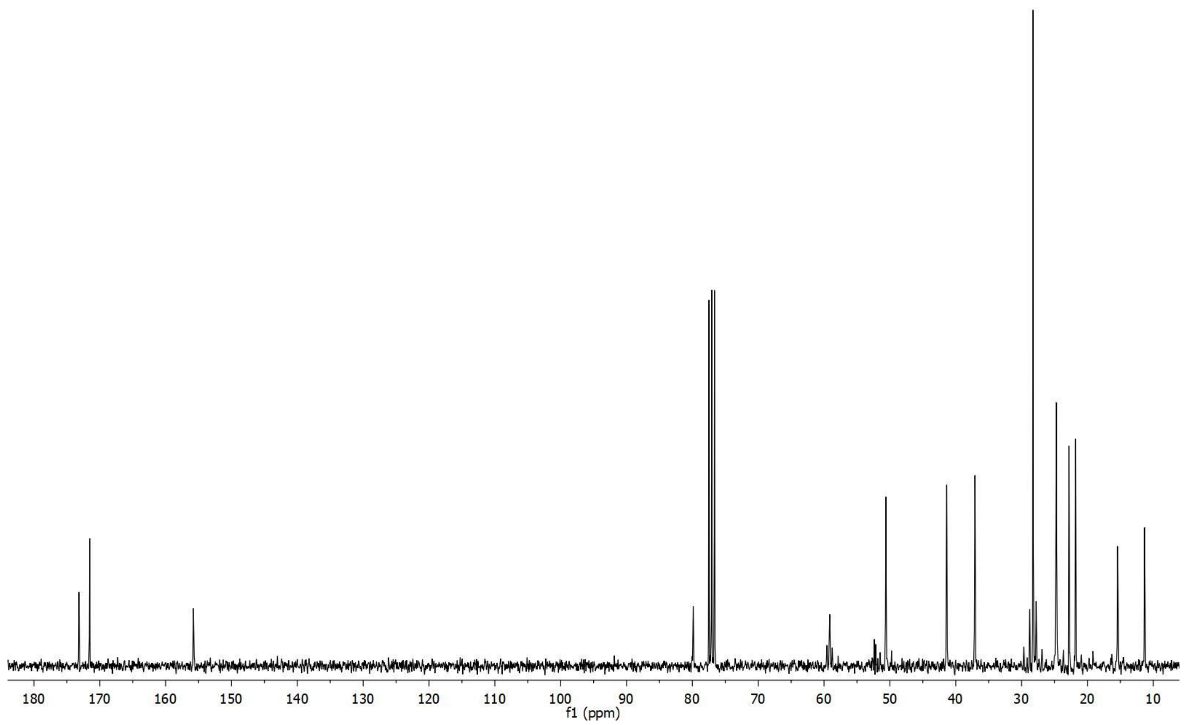

180
170
160
150
140
130
120
110
100
90
80
70
60
50
40
30
20
10
f1 (ppm)

MS (EI)

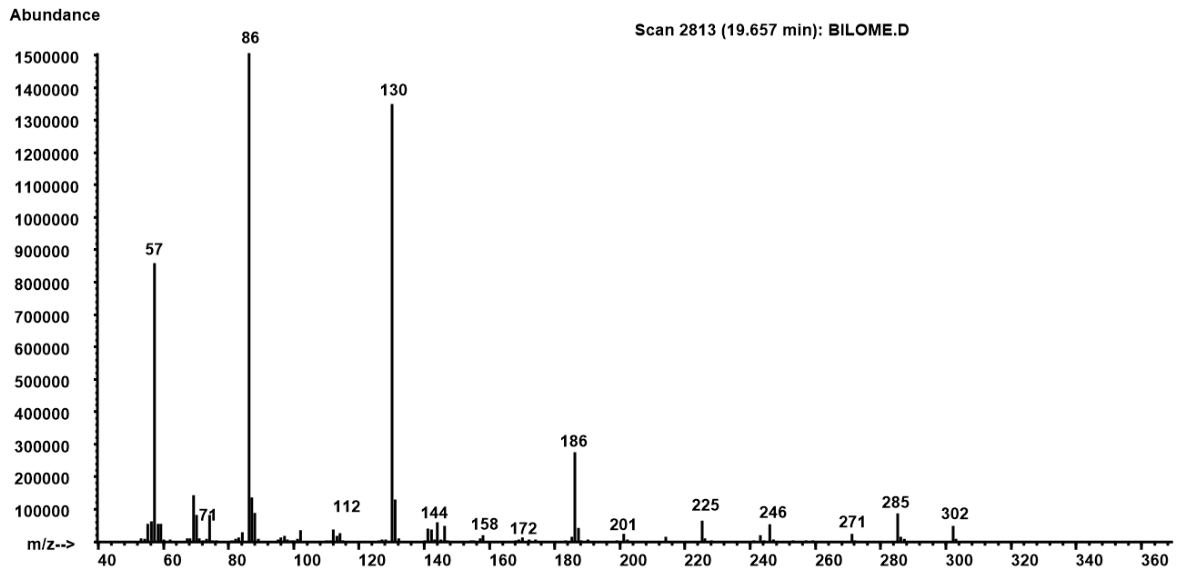

Abundance
Scan 2813 (19.657 min): BILOME.D
86
130
57
186
71
112
144
158
172
201
225
246
271
285
302
1500000
1400000
1300000
1200000
1100000
1000000
900000
800000
700000
600000
500000
400000
300000
200000
100000
m/z-->
40
60
80
100
120
140
160
180
200
220
240
260
280
300
320
340
360

N-Boc-L-Leu-L-Val-OMe (5b)

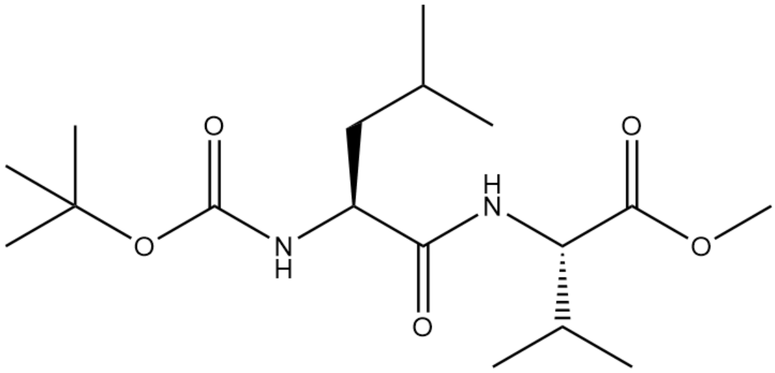

^{1}H NMR

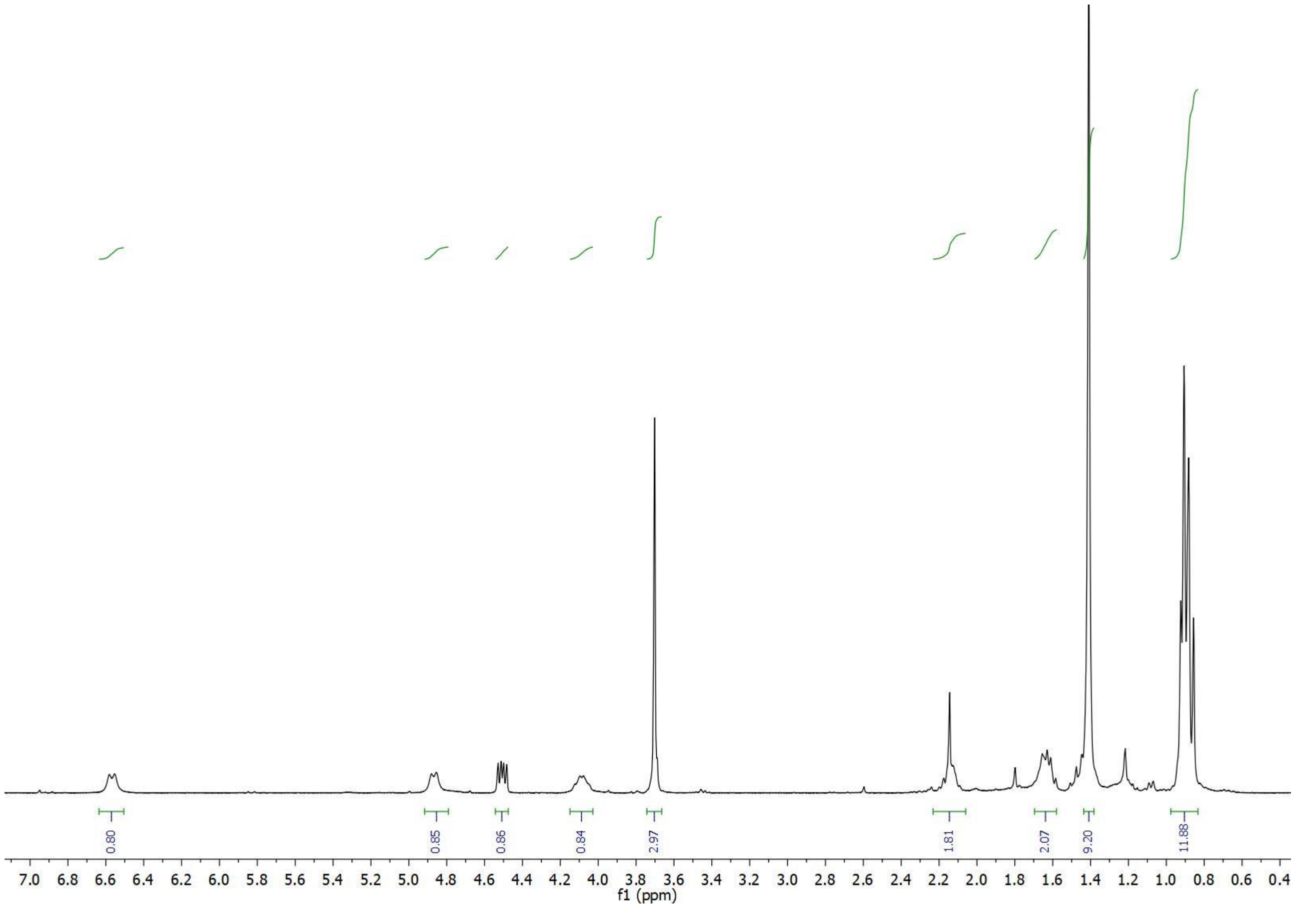

^{1}H NMR (300 MHz, $CDCl_3$) δ 6.57 (d_{broad}, *J* = 8.0 Hz, 1H, CONH), 4.87 (d, *J* = 8.2 Hz, 1H, OCONH), 4.51 (dd, *J* = 8.0, 4.9 Hz, 1H, C<u>H</u>COOMe), 4.09 (m, 1H, C<u>H</u>CONH), 3.70 (s, 3H, OCH_3), 2.21-2.07 (d, *J* = 4.6 Hz, 2H, C<u>H</u>$_2$CH(CH_3)$_2$, C<u>H</u>(CH_3)$_2$), 1.73-1.58 (m, 2H, C<u>H</u>$_2$C<u>H</u>(CH_3)$_2$), 1.41 (s, 9H, C(C<u>H</u>$_3$)$_3$), 0.97-0.83 (m 12H, CH(C<u>H</u>$_3$)$_2$, CH_2CH(C<u>H</u>$_3$)$_2$).

^{13}C NMR

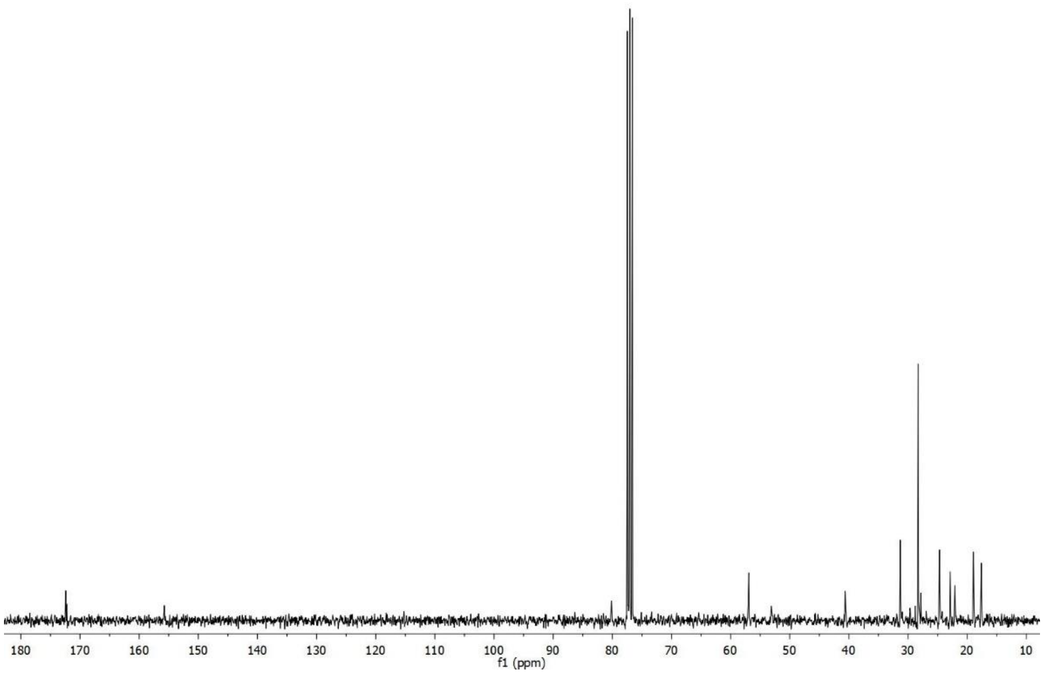

180
170
160
150
140
130
120
110
100
90
80
70
60
50
40
30
20
10
f1 (ppm)

MS (EI)

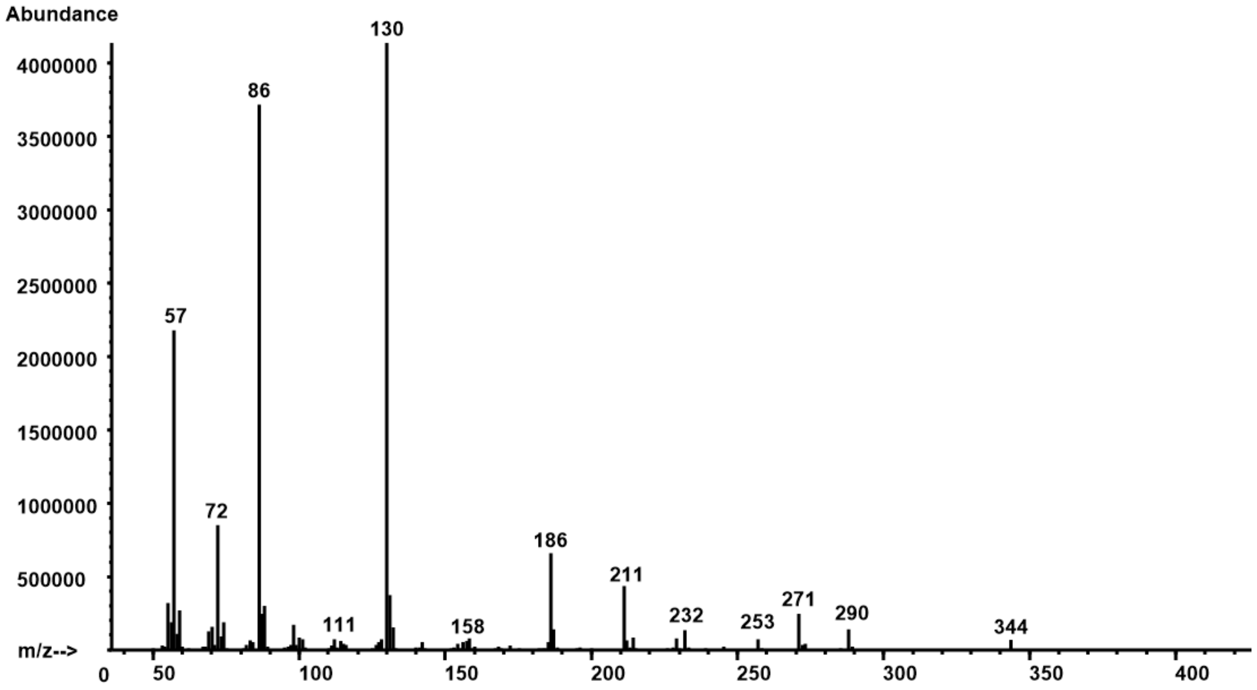

Abundance
4000000
3500000
3000000
2500000
2000000
1500000
1000000
500000
m/z-->
0
50
100
150
200
250
300
350
400
130
86
57
72
186
211
111
158
232
253
271
290
344

N-Boc-L-Arg(Z)$_2$-L-Ala-OMe (6b)

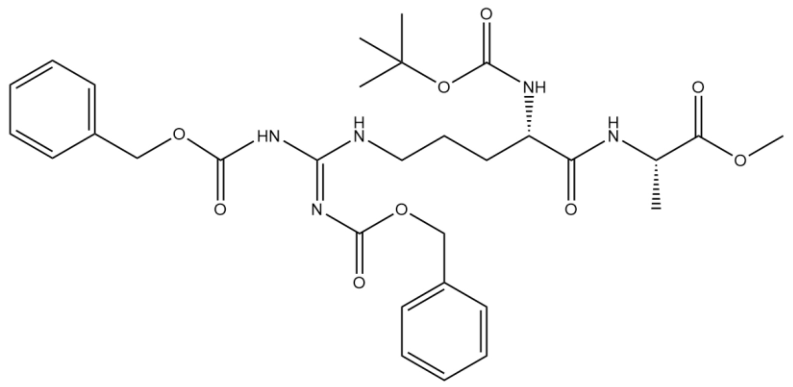

^{1}H NMR

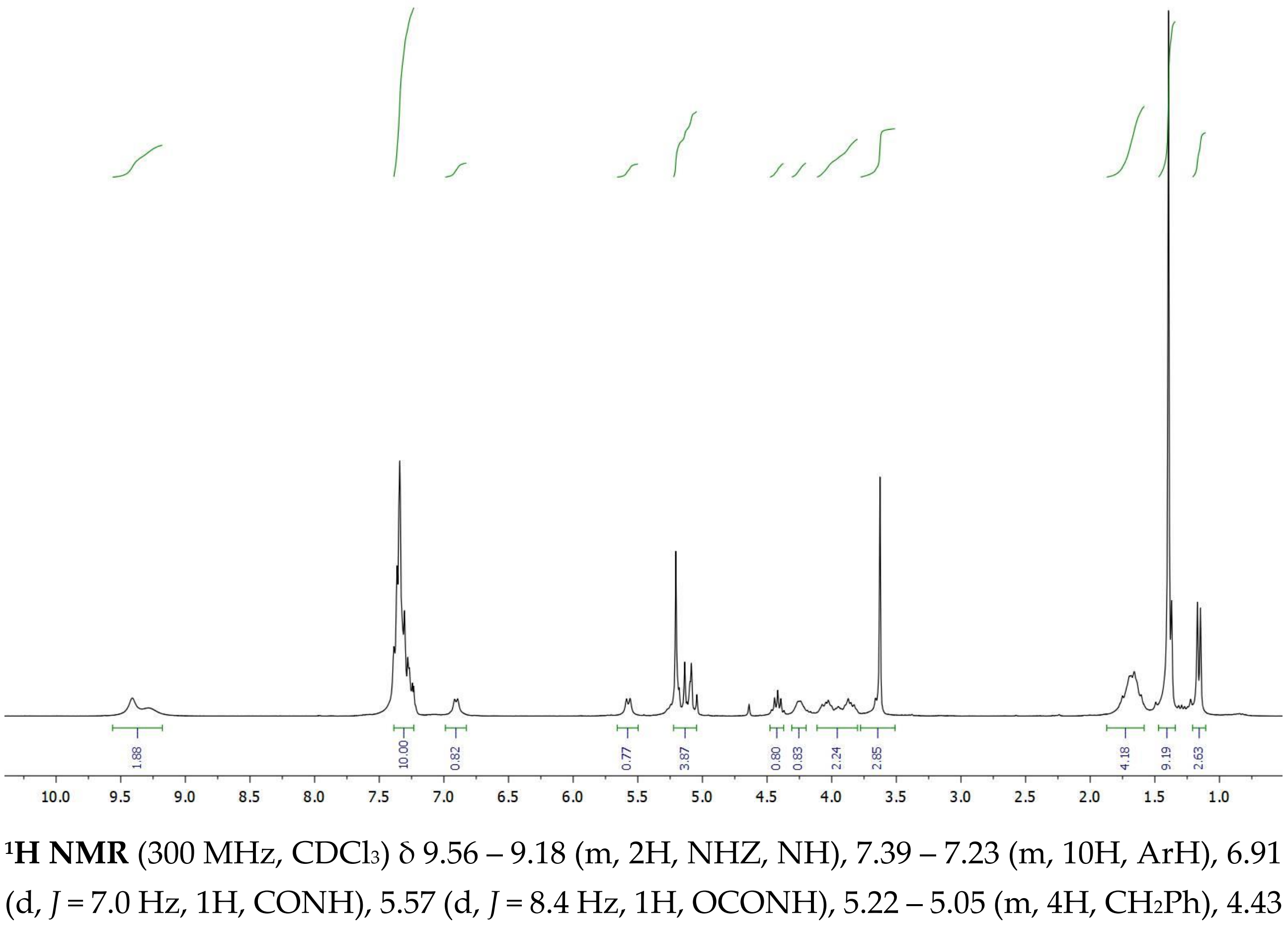

^{1}H NMR (300 MHz, $CDCl_3$) δ 9.56 – 9.18 (m, 2H, NHZ, NH), 7.39 – 7.23 (m, 10H, ArH), 6.91 (d, *J* = 7.0 Hz, 1H, CONH), 5.57 (d, *J* = 8.4 Hz, 1H, OCONH), 5.22 – 5.05 (m, 4H, CH_2Ph), 4.43 (m, 1H, C<u>H</u>COOMe), 4.25 (m, 1H, C<u>H</u>CONH), 4.12 – 3.75 (m, 2H, C<u>H</u>$_2$NH), 3.63 (s, 3H, OC<u>H</u>$_3$), 1.82-1.55 (m, 4H, CHC<u>H</u>$_2$C<u>H</u>$_2$CH$_2$NH), 1.39 (s, 9H, C(C<u>H</u>$_3$)$_3$), 1.16 (d, *J* = 7.2 Hz, 3H, CHC<u>H</u>$_3$).

^{13}C NMR

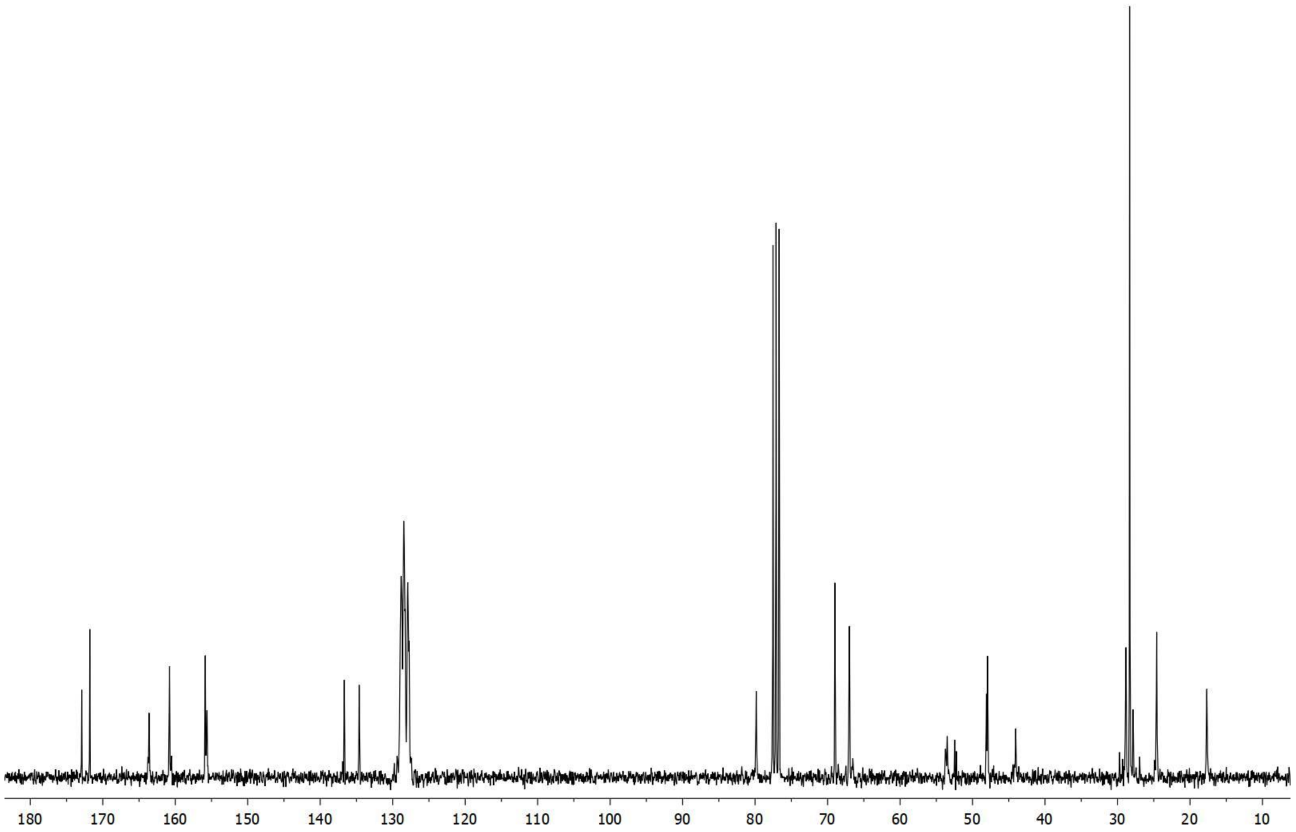

N-Boc-L-Phe-L-Leu-OMe (7b)

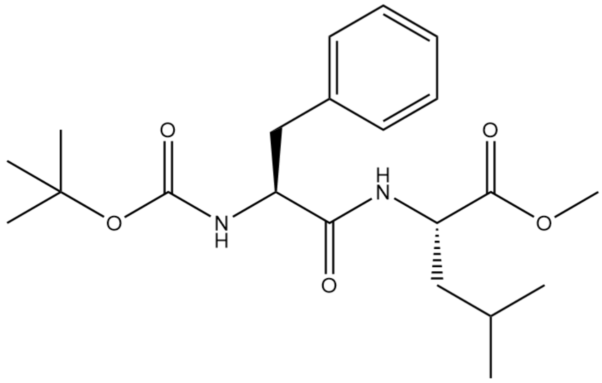

^{1}H NMR

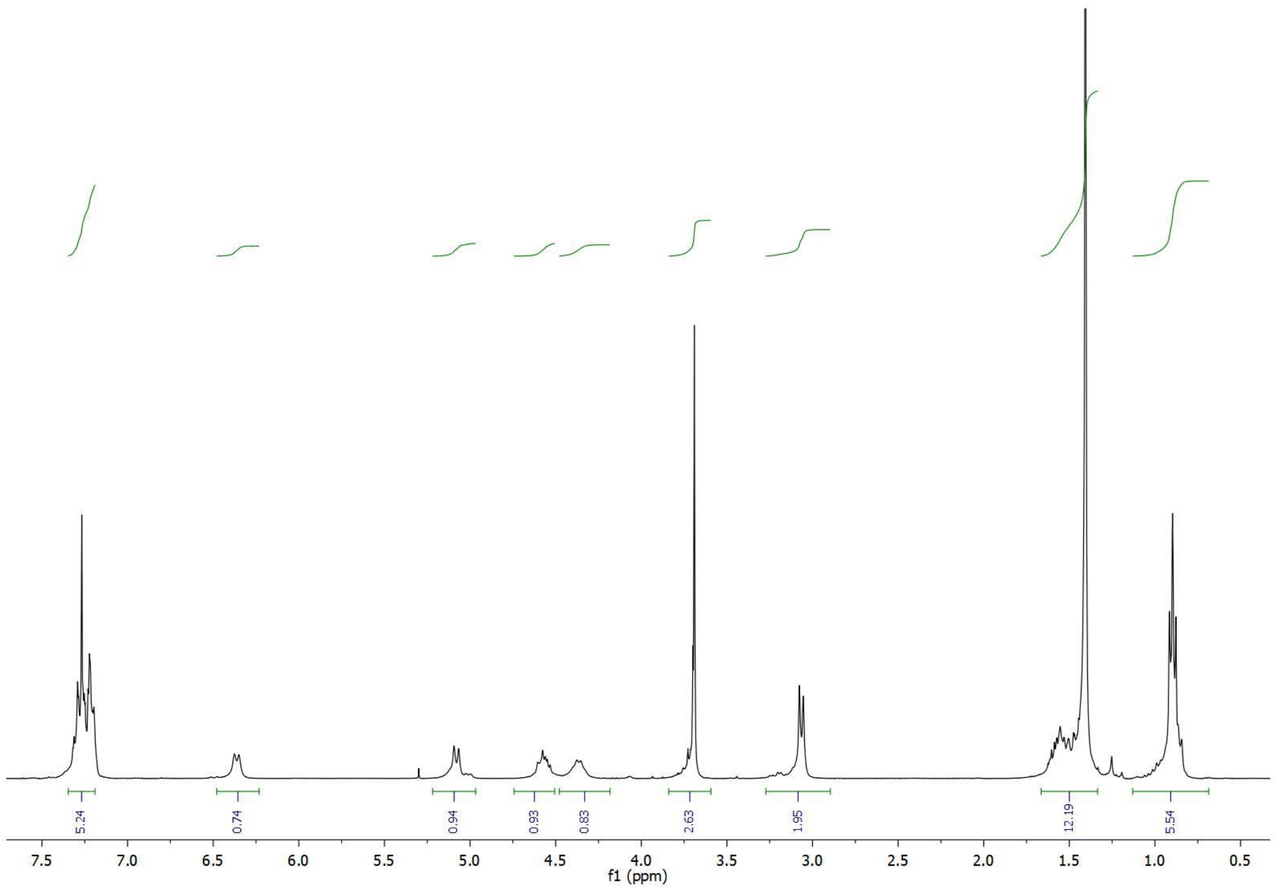

^{1}H NMR (300 MHz, $CDCl_3$) δ 7.44 – 7.09 (m, 5H, ArH), 6.36 (d, J = 7.9 Hz, 1H, CONH), 5.08 (d, J = 8.0 Hz, 1H, OCONH), 4.57 (m, 1H, CHCOOMe), 4.36 (m, 1H, CHCONH), 3.69 (s, 3H, OCH_3), 3.07 (d, J = 6.7 Hz, 2H, $CHCH_2Ph$), 1.66 – 1.33 (m, 12H, $CH_2CH(CH_3)_2$, $C(CH_3)_3$), 1.03-0.75 (m, 6H, $CH(CH_3)_2$).

^{13}C NMR

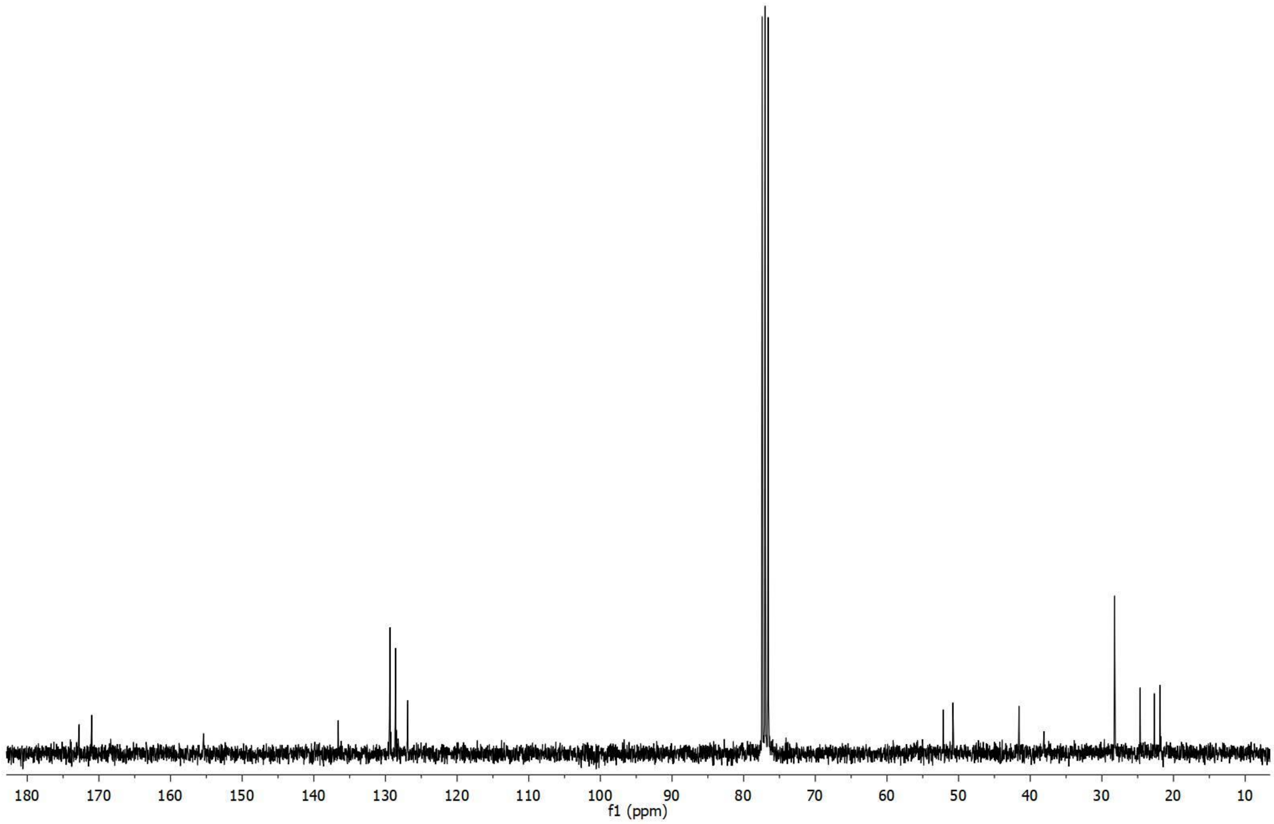
180
170
160
150
140
130
120
110
100
90
80
70
60
50
40
30
20
10
f1 (ppm)

N-Z-L-Ala-L-Ala-OMe (1c)

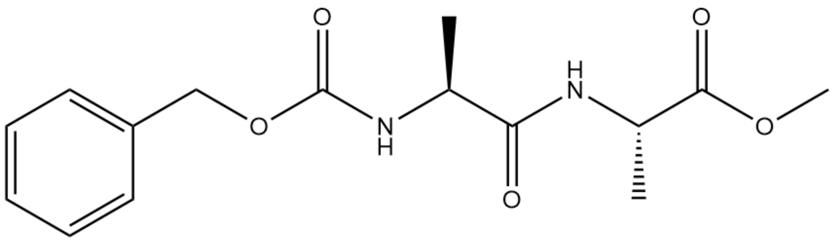

^{1}H NMR

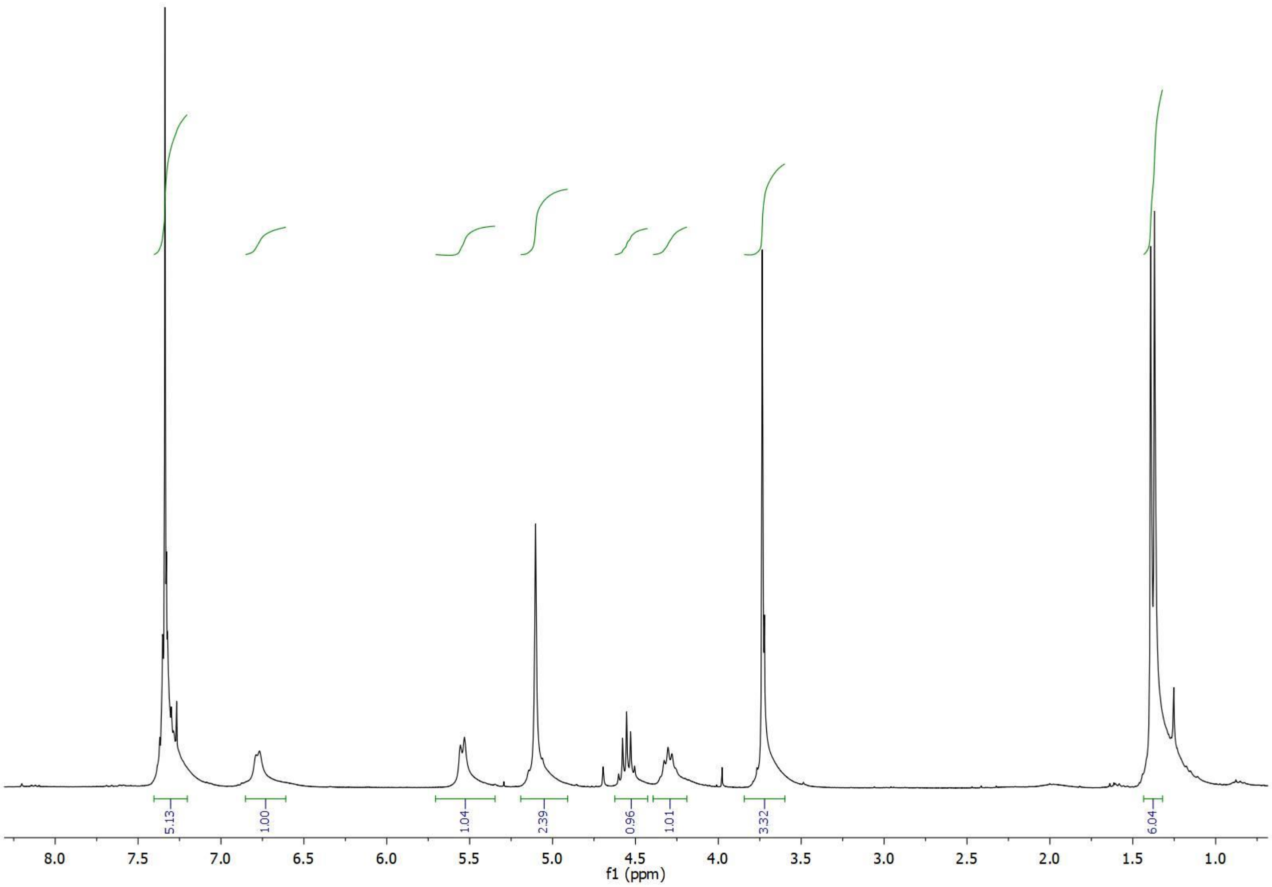

^{1}H NMR (300 MHz, CDCl3) δ 7.40 – 7.20 (m, 5H, ArH), 6.78 (d, J = 6.2 Hz, 1H, CONH), 5.54 (d, J = 7.5 Hz, 1H, OCONH), 5.10 (s, 2H, CH_2Ph), 4.55 (m, 1H, C$\underline{H}$COOMe), 4.30 (m, 1H, C$\underline{H}CH_3$), 3.73 (s, 3H, OCH_3), 1.38 (d, J = 7.0 Hz, 6H, CH_3).

^{13}C NMR

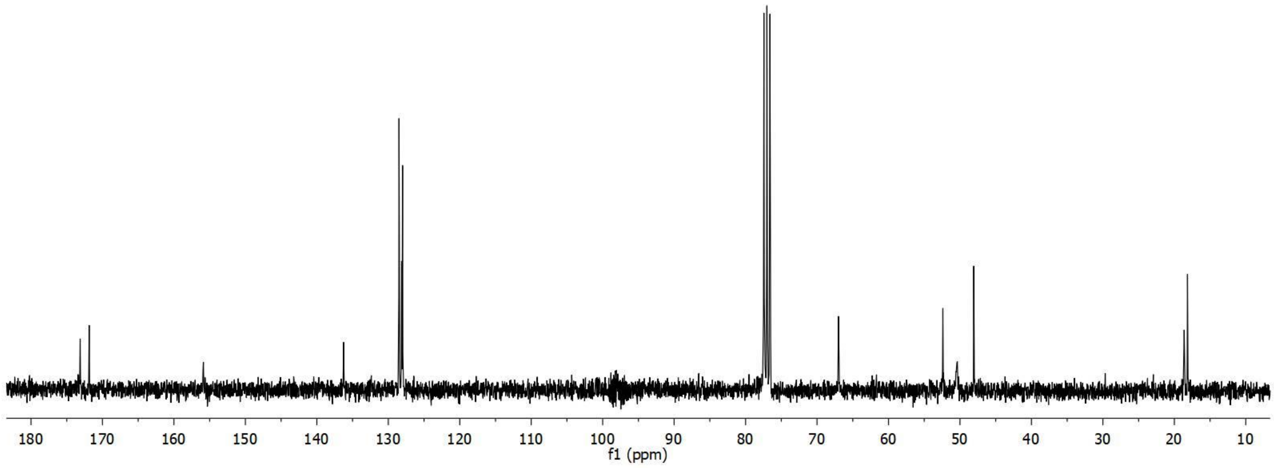

180
170
160
150
140
130
120
110
100
90
80
70
60
50
40
30
20
10
f1 (ppm)

MS (EI)

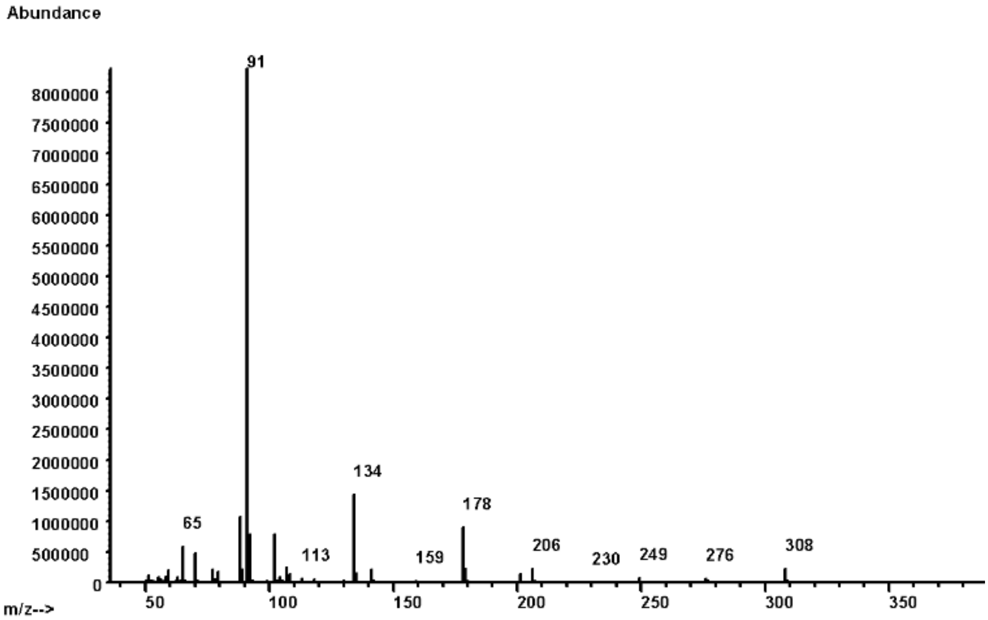

Abundance
91
134
178
65
113
159
206
230
249
276
308
8000000
7500000
7000000
6500000
6000000
5500000
5000000
4500000
4000000
3500000
3000000
2500000
2000000
1500000
1000000
500000
0
m/z-->
50
100
150
200
250
300
350

N-Z-Gly-L-Val-OMe (2c)

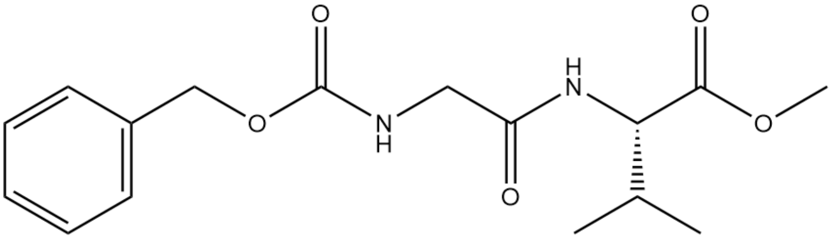

^{1}H NMR

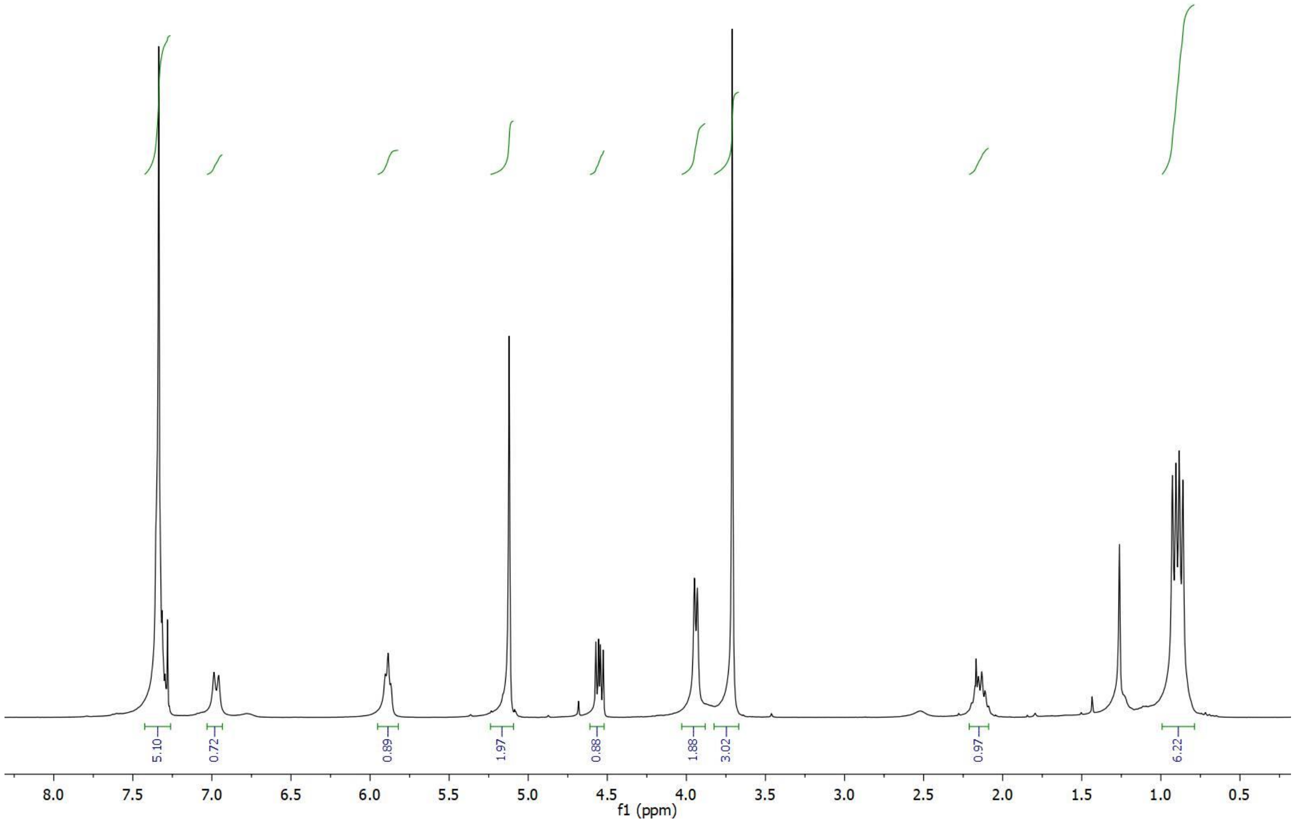

^{1}H NMR (300 MHz, $CDCl_3$) δ 7.42 – 7.26 (m, 5H, ArH), 6.97 (d, *J* = 8.8 Hz, 1H, CONH), 5.89 (m, 1H, OCONH), 5.12 (s, 2H, CH_2Z), 4.55 (dd, *J* = 8.8, 5.2 Hz, 1H, C$\underline{H}$COOMe), 3.94 (d, *J* = 5.5 Hz, 2H, C$\underline{H}_2$CONH), 3.71 (s, 3H, OCH_3), 2.21-2.09 (m, 1H, C$\underline{H}$(CH_3)$_2$), 1.03-0.77 (m, 6H, CH(C$\underline{H}_3$)$_2$).

^{13}C NMR

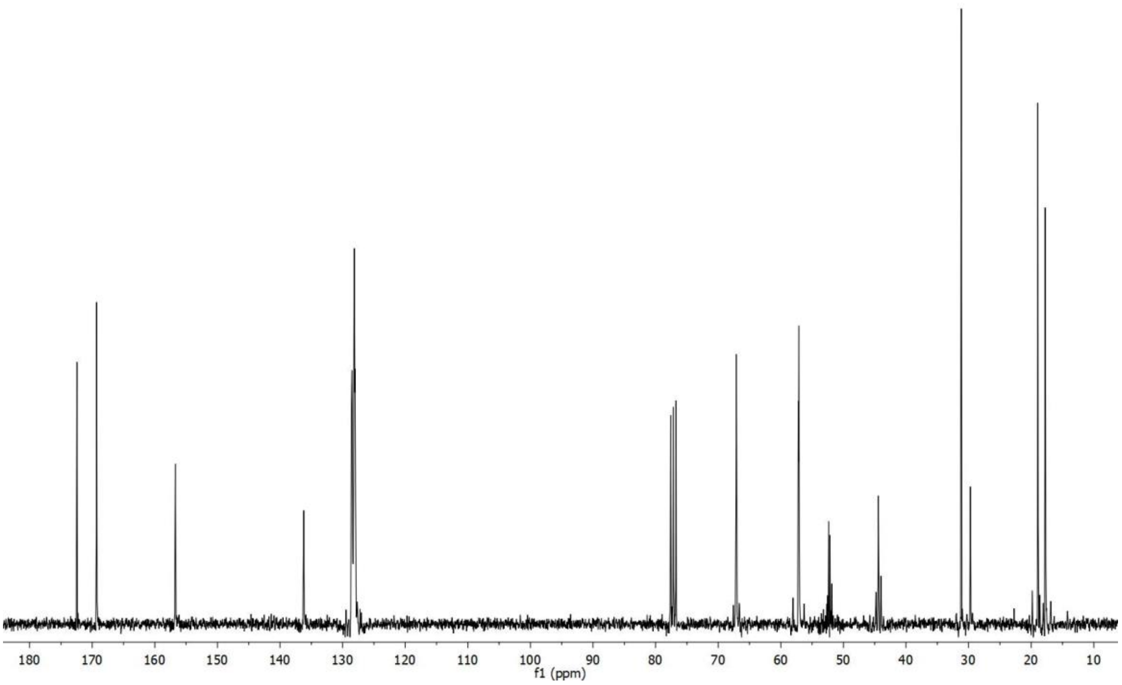

180
170
160
150
140
130
120
110
100
90
80
70
60
50
40
30
20
10
f1 (ppm)

MS (EI)

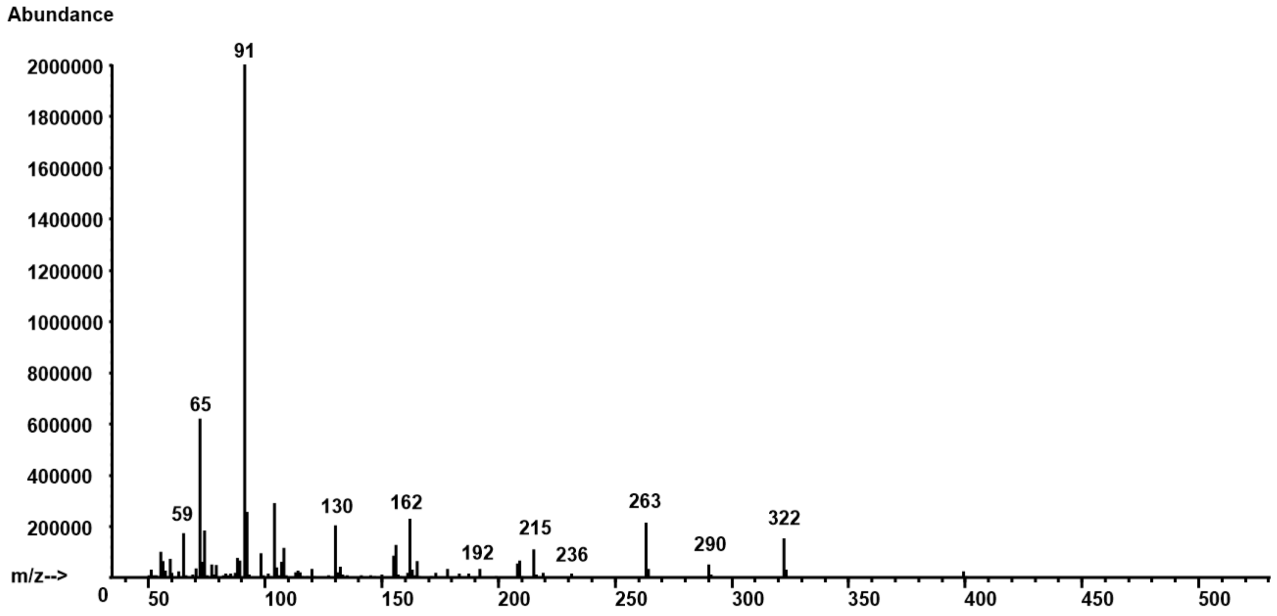

Abundance
2000000
1800000
1600000
1400000
1200000
1000000
800000
600000
400000
200000
m/z-->
0
50
100
150
200
250
300
350
400
450
500
91
65
59
130
162
192
215
236
263
290
322

N-Z-L-Val-L-Phe-OMe (3c)

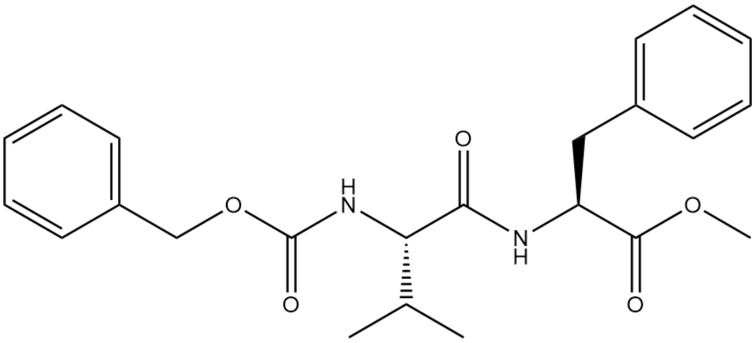

^{1}H NMR

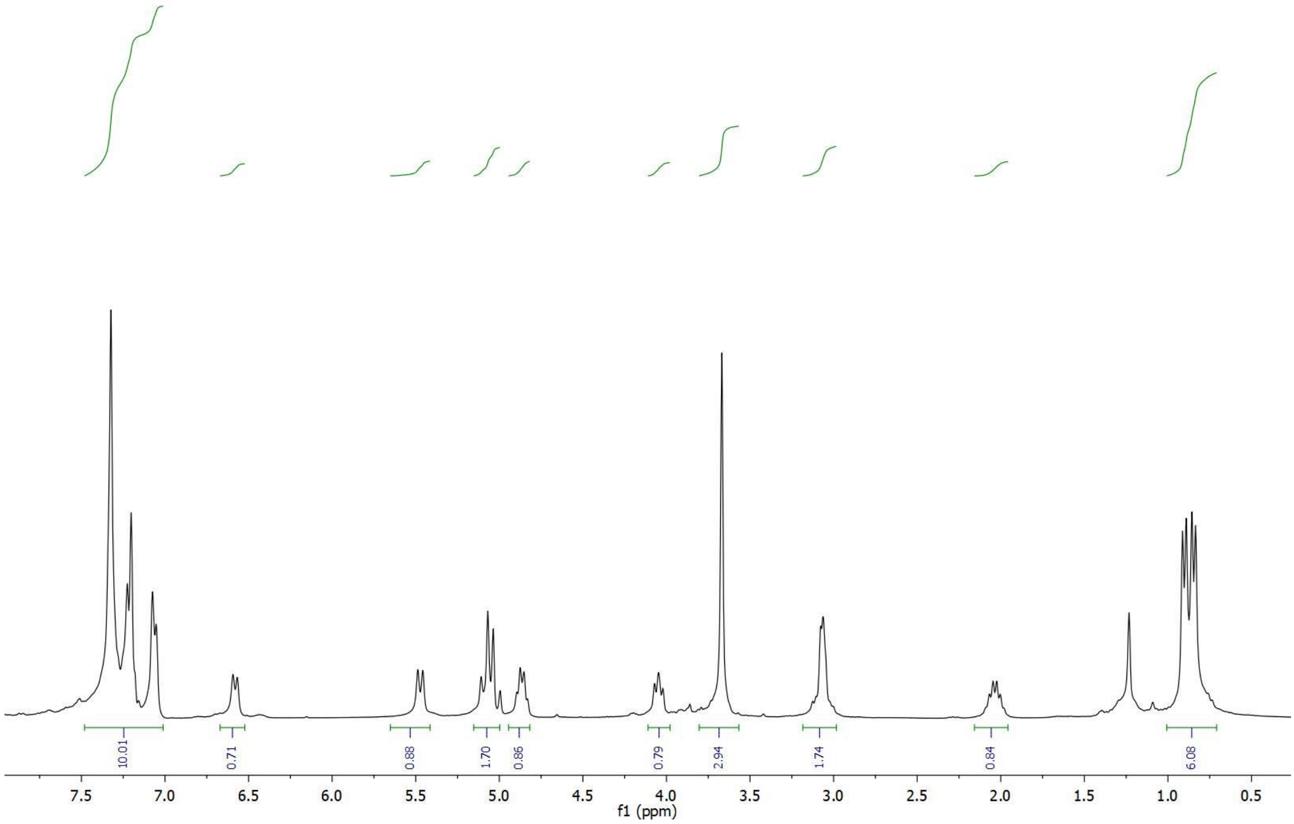

^{1}H NMR (300 MHz, $CDCl_3$) δ 7.51-7.00 (m, 10H, ArH), 6.58 (d, *J* = 7.6 Hz, 1H, CONH), 5.47 (d, *J*=8.8 Hz, 1H, OCONH), 5.16-4.96 (m, 2H, CH_2Z), 4.94-4.82 (m,1H, CHCOOMe), 4.11-3.98 (m, 1H, $CHCH(CH_3)_2$), 3.67 (s, 3H, OCH_3), 3.15-2.99 (m, 2H, CH_2Ph), 2.02 (m, 1H, $CH(CH_3)_2$), 0.97-0.73 (m, 6H, $CH(CH_3)_2$).

MS (EI)

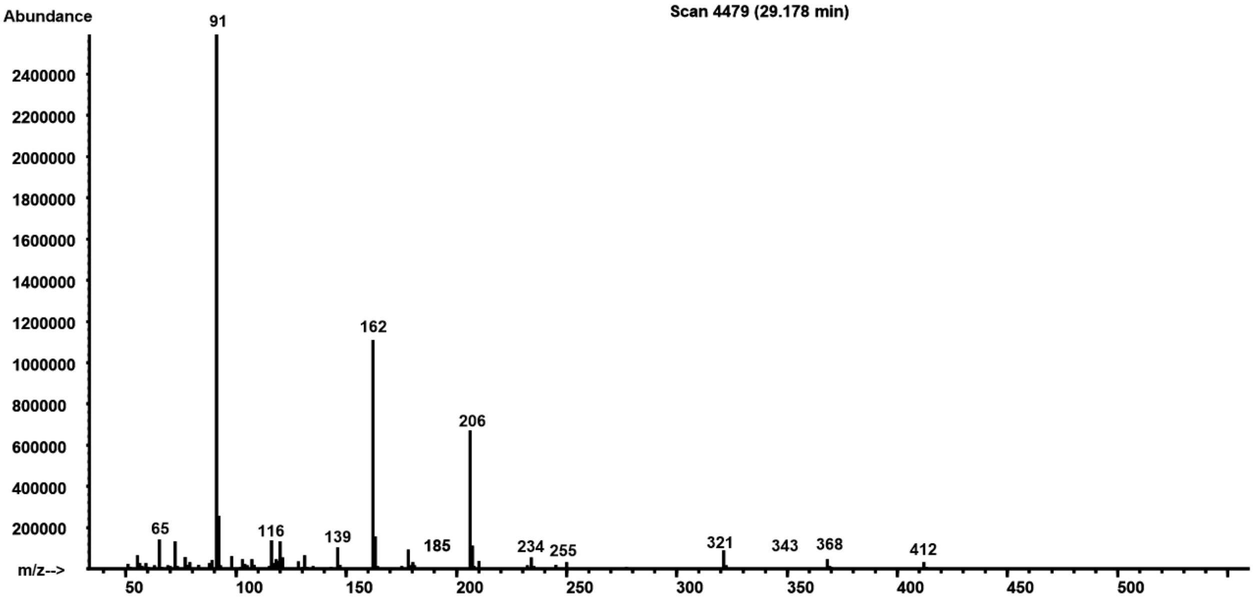

Abundance
Scan 4479 (29.178 min)
91
162
206
65
116
139
185
234
255
321
343
368
412
2400000
2200000
2000000
1800000
1600000
1400000
1200000
1000000
800000
600000
400000
200000
m/z-->
50
100
150
200
250
300
350
400
450
500
